# Pathogenesis-Driven Drug Repurposing with a Self-Nanoemulsifying Delivery System for Parkinson’s Disease

**DOI:** 10.3390/ph19081311

**Published:** 2026-08-20

**Authors:** Kunal Verma, Jaskiran Kaur, Mohit Kumar, Ankit Awasthi, Dinesh Kumar, Neeraj Choudhary, Emad M. Abdallah

**Affiliations:** 1 Chitkara College of Pharmacy, Chitkara University, Rajpura 140401, Punjab, India; vermakunal752@gmail.com (K.V.); jaskirankaur1092@gmail.com (J.K.); maddysharma3303@gmail.com (M.K.); 2 GNA School of Pharmacy, GNA University, Phagwara 144401, Punjab, India; dineshpotlia123@gmail.com (D.K.); dr.neerajchoudhary@gmail.com (N.C.); 3 Department of Biology, College of Science, Qassim University, Buraydah 51452, Saudi Arabia

**Keywords:** neurodegenerative disorder, Parkinson’s disease, microbiota, FDA-approved drug, blood–brain barrier, drug delivery

## Abstract

**Background/Objectives**: The aim of the present study was to investigate the mechanisms in Parkinson’s disease (PD), a progressive neurodegenerative disorder characterized by loss of dopaminergic neurons, aggregation of α-synuclein, mitochondrial dysfunction, oxidative stress, neuroinflammation, gut dysbiosis, and blood–brain barrier (BBB) impairment. Although there are several approved therapies that have been developed, their aqueous solubility, oral bioavailability, first-pass metabolism, and inability to penetrate the BBB make them less effective over time. This review is intended to critically analyze the potential of self-nanoemulsifying drug delivery systems (SNEDDSs) as a pathogenesis-related approach to enhance the delivery and therapeutic activity of repurposed drugs and conventional drugs for PD. **Methods**: A comprehensive literature search was conducted to address the pathogenic mechanisms of PD, the deficiencies of current pharmacotherapy, recent developments in SNEDDS formulation strategies and their application in improving oral bioavailability, lymphatic transport, BBB penetration and targeted brain delivery. A special focus was dedicated to drug repurposing, functionalized SNEDDSs, PEGylation, and gut–brain axis modulation. **Results**: SNEDDSs significantly enhance the water solubility, stability, intestinal absorption and systemic exposure of poorly water-soluble therapeutic agents and, to a certain extent, lymphatic uptake to avoid first-pass metabolism. These systems include improved brain delivery, decreased pharmacokinetic variability, and prolonged drug levels within the therapeutic range. Moreover, SNEDDSs can be used to deliver multiple molecules that are found to be neuroprotective, antioxidant, anti-inflammatory and probiotic, all at once, which can act on multiple pathogenic mechanisms associated with PD. Functionalized and PEGylated SNEDDSs add further to formulation stability, extend systemic circulation and increase efficiency of brain targeting. **Conclusions**: SNEDDSs are a promising translational nanomedicine platform for enhancing the effectiveness of conventional and repurposed therapeutics in PD, which address key pharmacokinetic and biological challenges. The next generation of oral therapies with targeted surface engineering, precision drug repurposing and clinical validation will be expected to bring about a faster advancement of drugs that can alter the course of disease rather than giving only symptomatic relief.

## 1. Introduction

Parkinson’s disease (PD) is a common and rapidly growing neurodegenerative disorder whose global prevalence continues to rise [1]. According to World Health Organization estimates, more than 8.5 million people were living with PD in 2019, and its prevalence had doubled over the preceding 25 years [2]. In the same year, PD accounted for approximately 5.8 million disability-adjusted life years and 329,000 deaths, representing increases of 81% and more than 100%, respectively, since 2000 [2].

PD is caused by the degeneration of dopamine neurons in the substantia nigra pars compacta (SNc) of the brain, and when dopaminergic neuronal death exceeds 50–60%, it affects motor function and leads to symptoms like bradykinesia, stiffness, postural instability, and resting tremors [3]. In addition to this, the gathering of misfolded protein α-synuclein in neurons leads to the intracellular aggregation of Lewy bodies, which is also responsible for PD. It has been proposed that the production of α-synuclein is responsible for neuroinflammation, neurotransmitter release impairment, and disturbance of the integrity of the blood–brain barrier (BBB) [4]. The majority of PD cases are caused by multiple factors, including genetic predisposition, aging, and environmental exposures, and other factors may also cause PD [5]. These factors impinge on dopaminergic neurons in the substantia nigra to produce progressive motor and non-motor symptoms. Additionally, mounting evidence indicates that α-synuclein pathology not only initiates neuronal degeneration but also enhances systemic and central immune responses, hence accelerating disease progression [6]. Furthermore, for the treatment of PD, out of all the delivery methods, oral delivery has emerged as the most popular treatment. However, drugs for PD are usually poorly soluble in water, have slow rates of dissolution, go through first-pass metabolism, and have various limitations. These factors ultimately result in low bioavailability and limited drug penetration into the brain [7]. Nanotechnology, an emerging beneficial tool, has addressed the above-discussed hurdles and enhanced the effectiveness of drugs by overcoming various factors [8,9]. Supportingly, some oral nanoformulations are already on the market and approved by the FDA, which improve brain targeting and bioavailability for the treatment of various PD symptoms.

Within the expanding landscape of drug delivery strategies investigated for PD, SNEDDSs represent a particularly compelling approach when evaluated against both conventional formulations and more complex nanotechnologies. Many advanced carrier systems, including polymeric nanoparticles, liposomes, and implantable or device-assisted platforms, face challenges related to formulation stability, manufacturing complexity, regulatory translation, or patient acceptability [10,11]. In contrast, SNEDDSs are simple, lipid-based systems that spontaneously generate nanoscale emulsions in the gastrointestinal environment, enabling efficient oral delivery of lipophilic, low-molecular-weight drugs that dominate current PD pharmacotherapy [12]. The present review provides an in-depth insight into the recent advancements in SNEDDSs for PD therapy. It also explores the integration of probiotics as an adjunct strategy to enhance gut–brain axis modulation and therapeutic outcomes. Furthermore, the review highlights the rationale for drug repurposing approaches that remain underexplored in the context of SNEDDSs, aiming to optimize formulation strategies for improved bioavailability and targeted delivery in PD management.

While this review provides a comprehensive overview of pathogenic mechanisms in PD and highlights the potential of SNEDDS-based oral delivery to support repurposed and conventional drugs, it is important to acknowledge that current evidence remains largely preclinical. The translational claims for SNEDDSs in PD are therefore prospective, and clinical validation is still lacking. Although several SNEDDS formulations are already marketed for other therapeutic indications, no PD-specific SNEDDS formulations have yet advanced to clinical trials. This underscores the need for rigorous pharmacokinetic and early-phase clinical studies to determine whether the improvements in solubility, bioavailability, and systemic exposure observed in vitro and animal models can be replicated in patients. By clarifying this limitation, we emphasize that SNEDDSs should be viewed as a promising but as yet unproven strategy for PD management, requiring careful translational research to establish their therapeutic value.

## 2. Key Pathogenic Mechanisms Involved in PD

**Figure 1:**
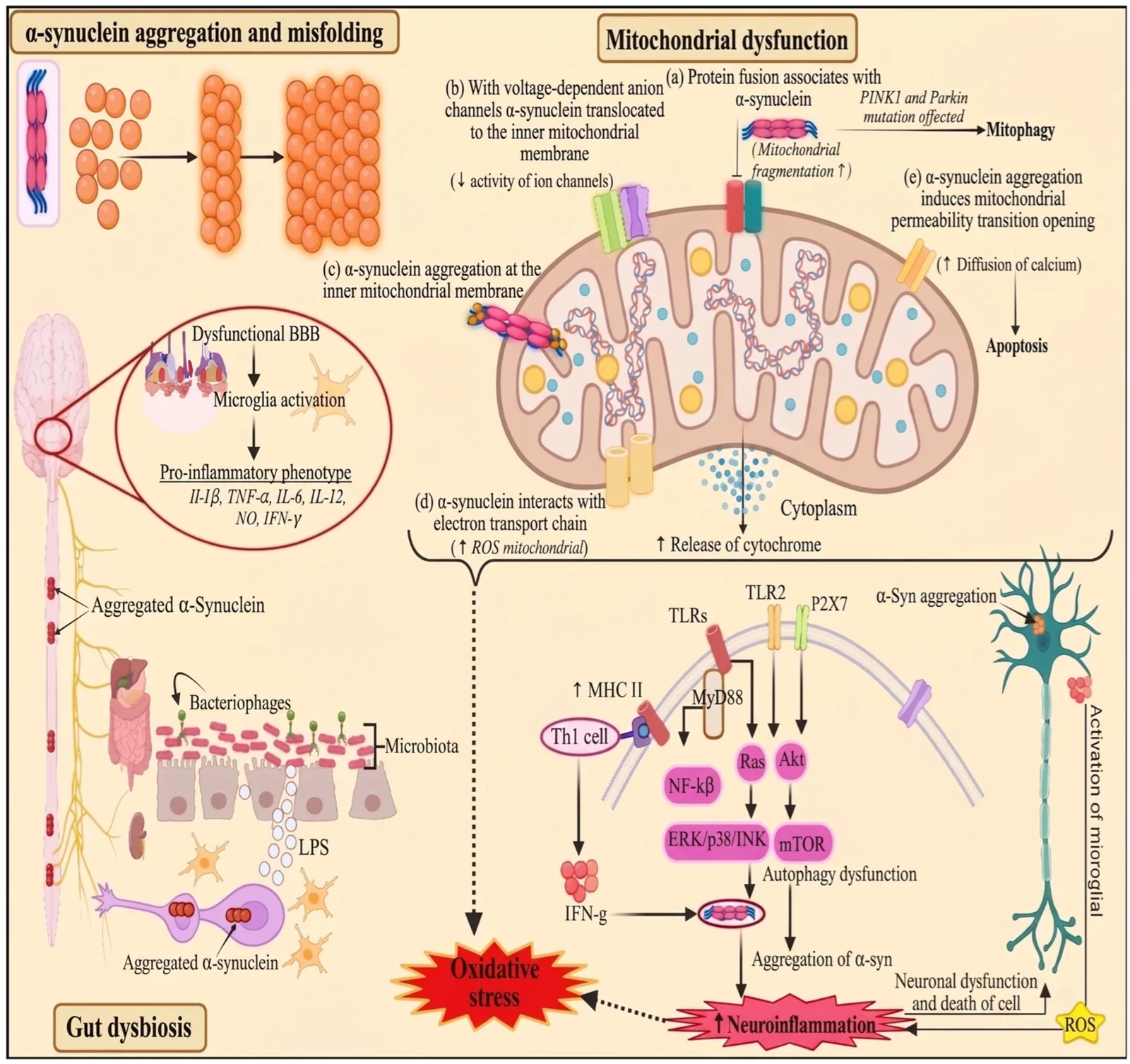
Integrative pathogenic mechanisms of PD: from α-synuclein aggregation to neurodegeneration.

The integrity of the BBB is essential for preserving the homeostasis of the brain, shielding it from harmful substances, which shows its protective properties and selective permeability, and promoting general brain health [13,14]. According to previous investigations, PD weakens the integrity of the BBB because of changes in tight junction and transporter protein levels, α-synuclein buildup, and an increase in inflammatory processes that cause blood molecules to extravasate, resulting in vascular degeneration. This might lead to a self-sustaining cycle of inflammation and a change in the BBB, where both processes support each other and cause neurodegeneration, eventually leading to PD [15]. This malfunction may facilitate access to the brain by inflammatory cells and neurotoxic agents, which, in turn, would cause neurodegeneration to become more severe and the symptoms of PD to be aggravated at a faster rate [16,17]. BBB disruption is not only indicative of an initial event in the development of PD; it also opens the door to various pathological mechanisms, like mitochondrial dysfunction, α-synuclein aggregation, oxidative damage, neuroinflammatory reaction, and the misregulation of the gut–brain axis, which in sum constitute PD, as discussed in detail and shown in Figure 1 [18].

### 2.1. α-Synuclein Misfolding and Aggregation

There are 140 amino acid residues that make up α-synuclein, which is a tiny cytoplasmic soluble protein, divided into three sections: the C-terminal domain, the non-amyloid-β component, and the N-terminal domain [19]. α-Synuclein has been the focus of intensive research for its biophysical and structural properties, as well as its pathophysiological role. Lewy neurites and Lewy bodies are the pathological features of PD, which are responsible for α-Syn aggregates [20]. Hence, it was suggested that α-synuclein aggregation is the key factor that leads to the advancement of PD [21]. In PD, α-synuclein aggregation is the core of the disease, which is certainly proven by point mutations in the gene encoding [22]. However, the relationship between the aggregation tendencies of α-synuclein mutants and the correlation with PD is not very clear. New research indicates that oligomers, which result from the early phases of the aggregation process, are the most toxic to cells, hence the death of the neurons in PD. Oligomers are diverse and unstable entities; thus, their characteristics are not well described [23]. α-Synuclein is an intrinsically disordered protein, and it has a great capacity for conformational plasticity by switching between a variety of conformations in different environmental conditions [24]. Recent research has shown that liquid–liquid phase separation (LLPS) contributes to the aggregation of α-synuclein, as reported in a study conducted by Xu et al., which examined how mutations associated with familial PD impact α-synuclein phase behavior. They inferred that E46K drives phase separation, whereas A30P, H50Q, E46K, and A53T facilitate amyloid aggregation. On the other hand, they also showed that the A53E mutation has the opposite effect and decelerates this process. These discoveries suggest that the LLPS event could signify a critical transition state and that mutation-specific aspects with respect to this stage may mediate PD pathology, as briefly discussed in Figure 1 [25].

### 2.2. Mitochondrial Dysfunction

Mitochondrial dysfunction is strongly implicated in the etiology of idiopathic and genetic PD. It is responsible for dopaminergic neuron death and, over time, it leads to PD [26]. The basic consequence of mitochondrial dysfunction is dysfunction in the electron transport chain (ETC), complex I, which increases oxidative stress, with resultant increased production of ROS and neuronal apoptosis by cytochrome C release [27]. Besides mitochondria-dependent apoptosis, other forms such as autophagy, necroptosis, ferroptosis, and pyroptosis also induce neuronal death [28]. Also, PINK1 and Parkin gene mutations compromise the mechanism of mitophagy, the selective breakdown of defective mitochondria, resulting in mitochondrial dysfunction, and these mutations are the cause of hereditary cases of PD [29]. PINK1 mutations impair preliminary recognition and signaling, causing damage to the mitochondria, as discussed in Figure 1. In contrast, Parkin mutations interfere with the amplification of the mitophagy signaling cascade, ultimately causing the build-up of nonfunctional mitochondria and activating the cell death of neurons [30]. In addition, α-synuclein, a main protein in synaptic function, aggravates mitochondrial malfunction at a toxic concentration or with mutated versions (A53T, A30P, G51D, E46K, and H50Q) [31]. Defective α-synuclein that links with mitochondria causes mitochondrial fission, oxidative stress, and an imbalance of calcium, which brings about further detriment to mitochondrial dynamics, followed by the apoptosis of the neuron [21].

### 2.3. Impairment of Protein Clearance

In PD, neurotoxic proteins like α-synuclein, amyloid-β (Aβ), and tau are major features of neurodegeneration. Dysfunction of protein clearance is a key factor in this process [32]. One of the major systems to remove these proteins is the glymphatic system, a brain-wide network for waste removal that enables the flow of cerebrospinal fluid (CSF) and interstitial fluid (ISF) through perivascular spaces (PVSs), with the help of astrocytes and their aquaporin-4 (AQP4) water channels [33]. In PD, the glymphatic system is not only structurally but also functionally impaired due to astrocytic damage, AQP4 mislocalization, and neuroinflammation. All these events result in a decrease in the brain’s capacity to detoxify neurotoxic proteins [34]. Notably, α-synuclein has increased deposition in glymphatic impairment, which has been confirmed in both autopsies of PD brains and animal models [35]. Deletion and malfunction of AQP4 in mice impairs the removal of alpha-synuclein, resulting in its aggregation, accelerated neuronal damage, and increased behavioral deficits. At the same time, clearance of both tau and Aβ is reduced in glymphatic dysfunction, resulting in the spreading of the neurodegenerative process and a decline in cognitive function [36,37]. Besides that, glymphatic dysfunction and protein aggregation are also mutually dependent, i.e., the occurrence of toxic proteins gives rise to further glymphatic dysfunction that ultimately results in the formation of a vicious cycle, which is the main factor [37]. This growing evidence suggests that impaired glymphatic clearance is the principal factor for proteinopathy and neurodegeneration in PD [38].

### 2.4. Neuroinflammation

CNS innate immune cells, mainly astrocytes and microglia, continuously activate and produce pro-inflammatory cytokines like interleukin-6 (IL-6), interleukin-1 beta (IL-1β), and tumor necrosis factor alpha (TNF-α). This is a key pathogenic mechanism in PD and is referred to as neuroinflammation [39].

Cytokines such as IL-6 and IL-1β are certainly indicators of systemic inflammation; however, they are the main culprits of continued neuroinflammation, which results in neuronal injury such as necroptosis, mitochondrial dysfunction, and synaptic transmission disruption [40]. Anti-inflammatory cytokines like IL-10 have been reported to be neuroprotective in their action by suppressing pro-inflammatory responses and inducing microglial homeostasis [41]. Misfolded and aggregated α-synuclein, which is an indicator of PD protein, is a powerful activator of microglia [37]. When α-synuclein is released into the extracellular environment, it binds with toll-like receptors (TLRs) on microglia, thus initiating NF-κB and other inflammatory signaling pathways and ultimately leading to elevated cytokine production [42,43]. Consequently, these neurons progressively degenerate, clinically presenting as bradykinesia, stiffness, and tremors, which are typical motor signs of PD, as shown in Figure 1. Chronic activation of microglia and astrocytes disrupts BBB integrity through cytokine-mediated downregulation of tight junction proteins and increased vascular permeability, leading to heterogeneous and unpredictable drug penetration into the central nervous system. This hostile inflammatory–oxidative microenvironment not only exacerbates neurodegeneration but can also reduce drug stability, alter transporter expression, and promote rapid clearance or subtherapeutic brain exposure [44]. Consequently, neuroinflammation and oxidative stress act as interconnected barriers that limit sustained pharmacological benefit, highlighting the need for delivery strategies capable of stabilizing drug exposure while simultaneously modulating inflammatory and oxidative pathways.

### 2.5. Oxidative Stress

One major contributing factor to the development of PD is oxidative stress that results in a discrepancy between the body’s ability to detoxify harmful molecules and the generation of reactive oxygen species (ROS), with the help of antioxidant defenses [45]. The main cause of dopaminergic neuron death is oxidative stress. This is because an oxidation–antioxidation disturbance causes excessive production of ROS, like hydroxyl radicals, superoxide anions, and hydrogen peroxide, which lead to DNA damage [46]. Under oxidative stress, matrix metalloproteinase 3 (MPP-3) becomes active, and when MPP-3 is activated, microglia produce ROS, leading to the release of TNF-α and IL-1β. The Nrf2-ARE signaling pathway, which is a primary antioxidant defense system, is usually damaged in PD, thereby decreasing the expression of protective enzymes like catalase [47,48]. The oxidative metabolism of dopamine to reactive intermediates, such as aminochrome, dopamine o-quinone, and 5,6-indolequinone, is believed to play a pivotal role in the neurodegenerative processes underlying PD [49]. Among these, aminochrome is particularly neurotoxic, as it induces different pathological mechanisms, with amplification of mitochondrial dysfunction and oxidative stress [50].

Previous studies emphasize that ROS are byproducts of mitochondrial metabolism that include hydrogen peroxide, superoxide anions, and hydroxyl radicals. In PD, damage to catalase results in oxidative stress and contributes to neuronal damage, as shown in Figure 1 [51]. Moreover, Shokolenko et al. showed that mtDNA can degrade due to oxidative stress, and in ROS-damaged mtDNA, strand breaks and basic sites predominate over mutagenesis base lesions [52]. Oxidative stress-related molecules like coenzyme Q10, DJ-1, uric acid, homocysteine, 8-hydroxy-2′-deoxyguanosin, vitamin E, retinoic acid/carotenes, superoxide dismutase, glutathione peroxidase, xanthine oxidase, and lipid peroxidation products may be potential PD biomarkers, as their altered levels may reflect oxidative imbalance and mitochondrial dysfunction [53,54].

Beyond its central role in dopaminergic neuron degeneration, oxidative stress also has important implications for drug delivery and therapeutic responsiveness in PD. These oxidative alterations create a hostile microenvironment in which conventional drugs exhibit reduced efficacy or shortened duration of action, particularly during long-term treatment. Consequently, strategies that combine antioxidant modulation with optimized drug delivery capable of maintaining stable therapeutic concentrations while mitigating oxidative damage are increasingly recognized as essential for improving treatment outcomes in PD.

### 2.6. Gut Dysbiosis

The structural and functional imbalance of the gut is known as one of the major pathogenic mechanisms of PD [55]. The vagus nerve is the most extensively dispersed and complex nerve, and the gut microbiota is the most prevalent and varied bacterial community in the human body, as illustrated in Figure 1. Both of these are necessary for preserving homeostasis [56]. Dysbiosis, or an imbalance in gut microbiota, can be responsible for inflammation and increased intestinal permeability, which can then negatively impact the CNS by encouraging α-synuclein aggregation and neuroinflammation [57]. Moreover, growing evidence indicates that PD might begin in the gut and subsequently spread to the CNS via the gut–brain axis, enteric nervous system, immune route, endocrine signals, and, most importantly, the bidirectional vagus nerve communication network [58]. In patients with PD, the gut microbiota exhibits significant dysregulation compared to healthy individuals, characterized by a marked reduction in short-chain fatty acid (SCFA)-producing and barrier-supporting bacterial genera such as *Faecalibacterium*, *Blautia*, *Roseburia*, and members of the *Prevotellaceae* family. This microbial imbalance may compromise intestinal barrier integrity and contribute to systemic inflammation, potentially affecting neurodegenerative processes by the gut–brain axis [55]. More recently, Leta et al. conducted a multicenter, randomized, double-blind, placebo-controlled trial involving 74 patients with PD and constipation. In the active treatment group, 12 weeks of a four-strain probiotic comprising *Lactobacillus acidophilus*, *Lactiplantibacillus plantarum*, *Lacticaseibacillus rhamnosus*, and *Enterococcus faecium* was associated with a shorter time-to-on after levodopa and a lower non-motor symptom burden, particularly fatigue and constipation; a between-group analysis also indicated a reduction in plasma TNF-α. However, MDS-UPDRS motor scores did not change significantly. These findings support further investigation of probiotics as adjunctive interventions for selected gastrointestinal and non-motor manifestations of PD but do not yet establish a broad improvement in motor symptoms [59]. Surprisingly, Hill-Burns et al. demonstrated that PD patients had higher amounts of *Bifidobacterium* and *Lactobacillus* than healthy individuals. This may be a regulatory response to compensation, but the exact mechanism is still unclear and not specified [60]. Such probiotic bacteria are essential for preserving the intestinal barrier’s integrity, balancing inflammation, and producing metabolites that protect the brain. If these probiotic bacteria are negatively impacted by killing, which is the main cause of the decrease in SCFA levels that negatively affect the integrity of gut barriers, a “leaky gut” state is generated. The leaky gut condition means that more toxins from deteriorated microbes can get into the bloodstream [61,62]. LPS is a potent endotoxin that has the ability to activate Toll-like receptor-4 (TLR4) on immune cells, inducing systemic inflammation and neuro-inflammatory responses in the brain. This neuroinflammation also leads to the degeneration of the substantia nigra’s dopaminergic neurons, which is common in PD [63,64]. In addition, gut dysbiosis directly influences α-synuclein misfolding and aggregation, a protein that abnormally misfolds in PD to create Lewy bodies. Misfolded α-synuclein can also be communicated from the gut to the brain through a prion-like mechanism, along with the vagus nerve, and can also be transmitted from the gut. Other factors like diet, lifestyle, and antibiotics also influence the gut microbiota and thereby increase PD risk. For instance, Western diets that are high in saturated fats and low in fiber can contribute further to dysbiosis [65]. Experimental evidence based on animal models has shown that the modulation of gut microbiota by antibiotics, probiotics, or fecal microbiota transplantation could affect PD-related pathology and symptoms [66]. Collectively, these observations highlight the central role played by gut dysbiosis in PD as etiologically pertinent and a prospective target for the development of new therapeutic interventions for restoring microbial homeostasis and avoiding neurodegeneration (Figure 1) [67].

## 3. Challenges of Conventional Therapy Against Parkinson’s Disease

Despite impactful progress in our understanding and handling of PD, conventional drug therapies, especially oral dopaminergic agents and those that are adjunctive, have a great number of challenges, as listed in Table 1, which lead to less positive patient outcomes than would have been possible [68]. There are various drugs for the management of PD, such as levodopa, dopamine agonists, MAO-B inhibitors, and COMT inhibitors. These drugs are effective in relieving symptoms and improving quality of life; however, frequent long-term use is related to various limitations.

One of the major problems in the conventional treatment of PD is that efficiency changes with time, which is characteristic of the “wearing off” phenomenon, along with the emergence of motor complications such as dyskinesias. This is caused by both continuous neurodegeneration and the pharmacokinetic characteristics of oral drugs, which often give rise to variable absorption, poor bioavailability, and inconsistent crossing of the BBB [8]. Therefore, the progression of the disease eventually makes the drug work less effectively. Management of side effects is a very serious problem. Levodopa may lead to dyskinesia, hallucinations, and impulse control disorders [69]. These side effects are most likely to lead to drug regimen changes or the addition of a new drug, which, in turn, may aggravate the patient’s burden and increase the chance of drug–drug interactions. Besides this, traditional methods of treatment can only partially help or do not support the main non-motor symptoms of PD, which are cognitive impairment, autonomic dysfunction, or depression [70]. Therefore, satisfaction with the treatment is limited, and those who take care of patients feel that the quality is very low [71]. Additionally, the design of the regimen and the patient’s commitment to taking the medication are also very important issues. There is a recognized need for user-friendly, less frequent, and more tolerable oral formulations in PD [72]. However, most of the medications that are approved for PD are solid oral drugs that require multiple doses per day. This leads to poor adherence, especially in elderly populations that may have trouble swallowing or cognitive decline. In addition, the pharmacokinetic profiles of these drugs are often unpredictable due to factors like gastrointestinal motility changes and food interactions, which are common in advanced PD [73]. There are a great number of medications that exist, but none of them have been able to cure PD, providing symptomatic control instead of disease modification or neuroprotection [74]. These problems emphasize the need for the next generation of therapeutic interventions—including the use of advanced drug delivery like nanomedicine and neuroprotective drugs that can better meet the requirements of PD [75]. The advantages and challenges associated with traditional pharmacotherapy in Parkinson’s disease are given in Table 1.

**Table 1:** Advantages and challenges associated with traditional pharmacotherapy in Parkinson’s disease.

Advantages	Challenges	References
Alleviation of motor symptoms	Motor fluctuations and dyskinesia associated with prolonged usage	[76,77,78]
Enhanced quality of life	Reduced effectiveness over time	[76,79]
Delay of significant impairment	Persistence of non-motor symptoms (e.g., cognitive deterioration, affective disorders)	[77]
A diverse variety of available drugs	Negative consequences (e.g., nausea, hallucinations, impulse control issues)	[76,78]
Experiential therapy alternatives	Recurrent administration necessary (intricate schedules and medication burden)	[76,78]
Personalized dosing enhances symptom management and diminishes “off” periods	It may necessitate hospitalization or frequent modifications and depends on subjective reporting	[79]
Refinement through wearable sensors and instruments facilitates further customization	Access to technology and device compatibility may be restricted, necessitating training and compliance	[76]
Real-time medication optimization	Potential for data privacy violations and variable effectiveness among individuals	[78]
Cost-effectiveness	Restricted effectiveness for axial symptoms (e.g., balance, speech, freezing of gait)	[76]

In addition to this, FDA-approved drugs are deeply rooted in clinical practice and are actively used by patients to alleviate motor and non-motor symptoms, as listed in Table 2. These therapeutic drugs, including carbidopa/levodopa combinations (such as Sinemet, Duopa, and Rytary), dopamine agonists (like pramipexole, rotigotine, and ropinirole), MAO-B inhibitors (rasagiline, selegiline, and safinamide), COMT inhibitors (entacapone, opicapone, and tolcapone), and adjunctive treatments (such as amantadine, istradefylline, pimavanserin), are the primary means of evidence-based PD management [80]. All of these drugs have undergone the FDA’s rigorous clinical evaluation process, and their use in the human population is proven to be effective in relieving basic motor symptoms, such as tremor, bradykinesia, and rigidity [81]. Nevertheless, clinical experience and post-marketing surveillance have made it clear that the range of side effects caused by the drugs may complicate management and adversely affect a patient’s life [82]. To be more specific, levodopa-based therapies may give rise to manifestations of uncontrollable movements (dyskinesias, motor fluctuations) and be the cause of neuropsychiatric symptoms (hallucinations, impulse control disorders). Dopamine agonists are therefore linked to problems with edema and a higher danger of developing compulsive behaviors, whereas MAO-B and COMT inhibitors may be responsible for some patients’ gastrointestinal complaints, insomnia, and, in very rare cases, liver disease [83].

## 4. Rationale and Mechanistic Insight into SNEDDSs in PD

SNEDDSs have become one of the best nanocarriers for PD because they provide a simple formulation with highly effective oral delivery. Liposomes or cubosomes are unstable in the gastrointestinal tract and require complicated manufacturing processes, while SNEDDSs will automatically produce fine nanoemulsions upon contact with digestive fluids, thus ensuring very fast solubilization and even distribution of poorly soluble drugs like levodopa or dopamine agonists. This self-emulsification mechanism boosts the drug’s bioavailability, cuts down on the variability in the drug’s absorption, and results in higher drug plasma levels, which are essential for reducing the “on–off” motor fluctuations that are characteristic of PD patients. If we talk about pharmacosomes, the major difference from SNEDDSs is that they involve chemical conjugation of drugs to lipids, while SNEDDSs are more flexible and simpler to scale up for production. Invasomes and transferosomes are mainly designed for transdermal delivery and thus are less suitable for PD therapy, which needs regular oral dosing. Collidosomes and metallic nanoparticles are mostly at the research stage, with issues of brittleness, toxicity, and long-term safety; thus, they are not quite suited for lifelong treatment. Polymeric nanoparticles, along with micelles, can enhance drug targeting, but they are frequently confronted with problems, such as complicated synthesis, unstable products, and regulatory difficulties, whereas SNEDDSs employ well-characterized excipients that have been proven safe. Most importantly, SNEDDSs are capable of facilitating lymphatic transport, which also means partially bypassing first-pass metabolism and thus improving brain delivery of dopaminergic drugs, resulting in an increase in therapeutic efficacy even at lower doses and a decrease in drug side effects. As they are easy to administer, scalable, and able to provide sustained and predictable drug release, SNEDDSs are becoming a more patient-friendly and clinically feasible option for PD than other advanced nanocarrier systems. In Table 3, we discuss different nanoformulations, advantages, and challenges for PD and why SNEDDSs are important despite these challenges.

Oral drug delivery is confronted with numerous key drawbacks when delivered to the CNS. Firstly, PD drugs have low aqueous solubility and are poorly permeable, which heavily impedes their GI tract absorption [101]. Secondly, after absorption, much of the drug is subjected to extensive hepatic first-pass metabolism, significantly lowering its bioavailability before it can enter systemic circulation [102]. Thirdly, even if part of the drug enters the bloodstream, it is confronted with another difficult obstacle, the BBB, which prevents more than 98% of small molecules and almost all large molecules from entering the brain. These pharmacokinetic problems lead to the requirement of high doses, which tend to produce side effects and unfavorable therapeutic results [103]. The Biopharmaceutics Classification System classifies drugs according to their aqueous solubility and intestinal permeability. Classes II and IV are characterized by low aqueous solubility, while class IV additionally exhibits low permeability. Accordingly, improving dissolution and maintaining the drug in a solubilized state are important formulation goals, although solubility enhancement alone may not fully overcome the absorption limitations of class IV drugs [104,105].

SNEDDSs are an especially potent method of improving the oral delivery of lipophilic and poorly soluble PD drugs [12]. SNEDDSs are isotropic mixtures of oils, surfactants, and cosolvents that spontaneously form fine oil-in-water nanoemulsions following dilution and gentle agitation in gastrointestinal fluids. The resulting small droplets increase the interfacial surface area, helping to maintain the incorporated drug in a solubilized state and potentially enhancing its dissolution and intestinal absorption [105]. Most importantly, SNEDDSs allow for the association of lipophilic drugs with dietary lipids to form chylomicrons within enterocytes. These chylomicrons are absorbed through the intestinal lymphatic pathway, circumventing the liver and thus preventing first-pass metabolism. This pathway of lymphatic uptake leads to a significant enhancement in the systemic bioavailability of the drug, which can be circulated and access the CNS [106].

Comparative evidence with other nanocarriers further highlights the potential value of SNEDDSs for oral delivery of PD therapeutics, although direct head-to-head comparisons of the same active moiety across nanocarrier platforms remain unavailable. For morin, an oral morin–phospholipid complex-loaded SNEDDS produced a mean droplet size of approximately 140 nm, increased Cmax from 5.53 to 28.60 μg/mL, and achieved a 6.23-fold increase in relative oral bioavailability compared with a morin suspension [107]. In comparison, orally administered lipid-core/PLGA-shell morin nanoparticles (~200 nm, 82% loading efficiency) produced a 5.6-fold increase in bioavailability, with AUC increasing from 1294 ± 331 to 7306 ± 1609 ng·h/mL and plasma half-life from 0.13 to 0.98 h. Although these studies used different experimental conditions and therefore cannot establish superiority, the greater relative bioavailability reported with SNEDDSs suggests a strong advantage for improving the oral exposure of morin, whereas a PLGA system provided the additional benefit of sustained release [108]. A similar distinction is evident for ropinirole. Ropinirole-loaded PLGA nanoparticles showed 74.8 ± 8.2% encapsulation efficiency, a particle size below 155 nm, and approximately 5 days of zero-order drug release, and their parenteral administration produced beneficial behavioral and histological effects in rotenone-induced PD rats, including improvements in catalepsy, akinesia, rotarod and swim performance and preservation of tyrosine hydroxylase-positive neurons [109]. In contrast, an oral ropinirole–SNEDDS formulation resulted in a mean droplet size of 96.71 nm, an emulsification time of 22 s, and approximately 99% drug release compared with ~30% from the pure drug, demonstrating substantially improved formulation and release performance. The SNEDDS formulation was specifically developed to mitigate ropinirole’s extensive first-pass metabolism, with enhanced lymphatic uptake proposed as a mechanism to reduce hepatic exposure [110]. However, in vivo pharmacokinetic and PD efficacy data were not reported for this particular SNEDDS. Taken together, the available findings suggest that SNEDDSs may be particularly advantageous when the primary objective is oral solubilization, rapid dispersion, and improved systemic exposure, whereas polymeric nanoparticles may be better suited for sustained release and, in selected parenteral applications, direct CNS-targeted delivery. Future clinical studies are essential to validate these complementary roles and establish their comparative therapeutic value in PD.

**Figure 2:**
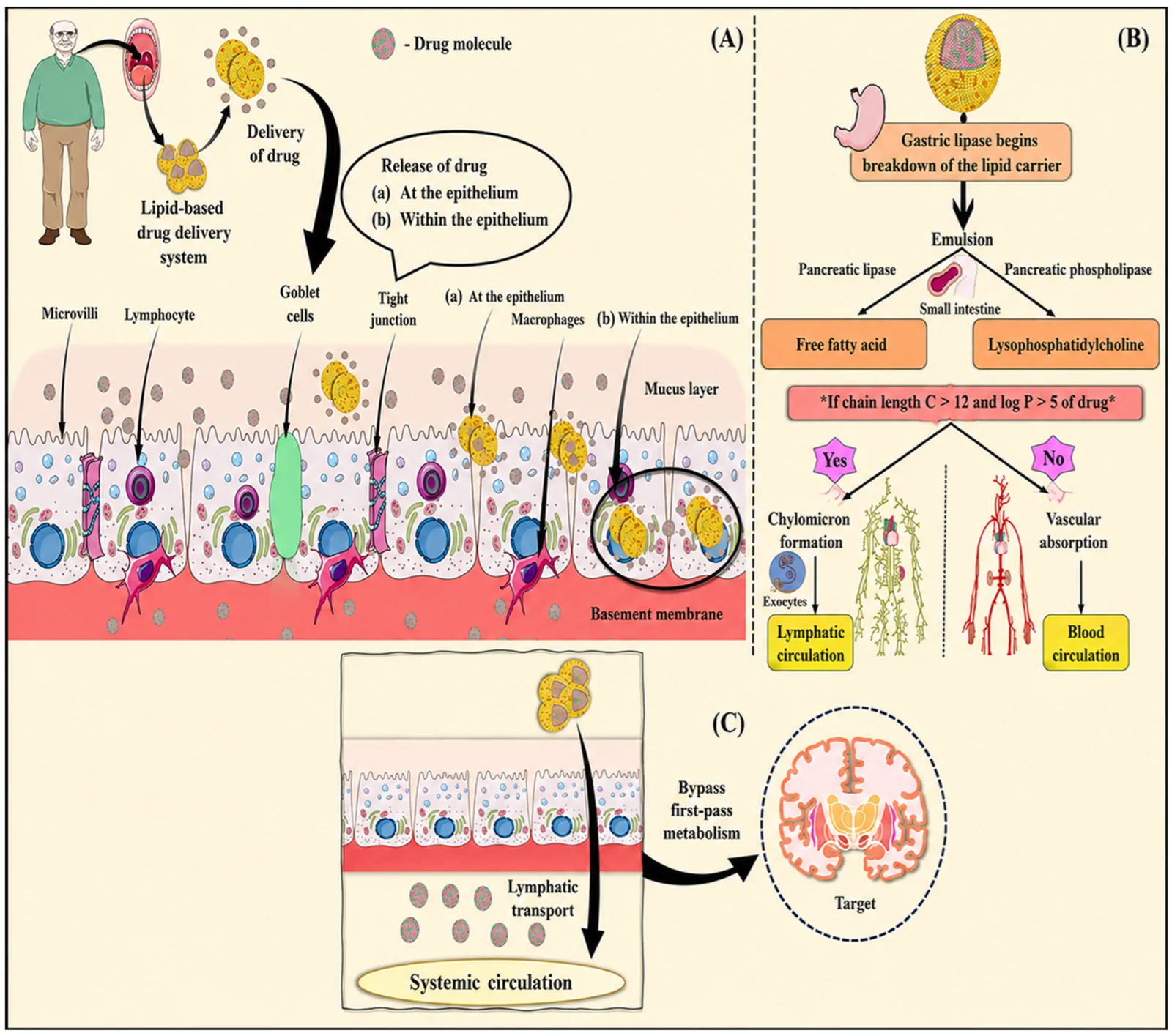
Illustration of drug-loaded SNEDDSs for targeting the brain with the help of the lymphatic route. (**A**) Oral administration and initial drug release, where the drug passes through the mucus layer of the gastrointestinal (GI) tract, exhibiting mucoadhesion, which ensures close contact with the epithelium and drug molecule release either at the epithelial surface or within the epithelial cell, after which absorption occurs via enterocytes. (**B**) Digestion and processing of lipid carriers: gastric lipase begins the breakdown of the lipid carrier in the stomach; then, enzymes like pancreatic lipase and phospholipase A2 further digest the lipids in the small intestine, which is responsible for generating free fatty acids and lysophosphatidylcholine. If a drug has high lipophilicity (log P > 5) and long lipid chains (C > 12), then it is directed towards lymphatic system absorption by chylomicron formation rather than the vascular route. (**C**) Lymphatic transport and systemic circulation: chylomicrons are exocytosed, enter the lymphatic transport, and reach the systemic circulation while bypassing first-pass metabolism; drug release occurs gradually at target organs.

**Figure 3:**
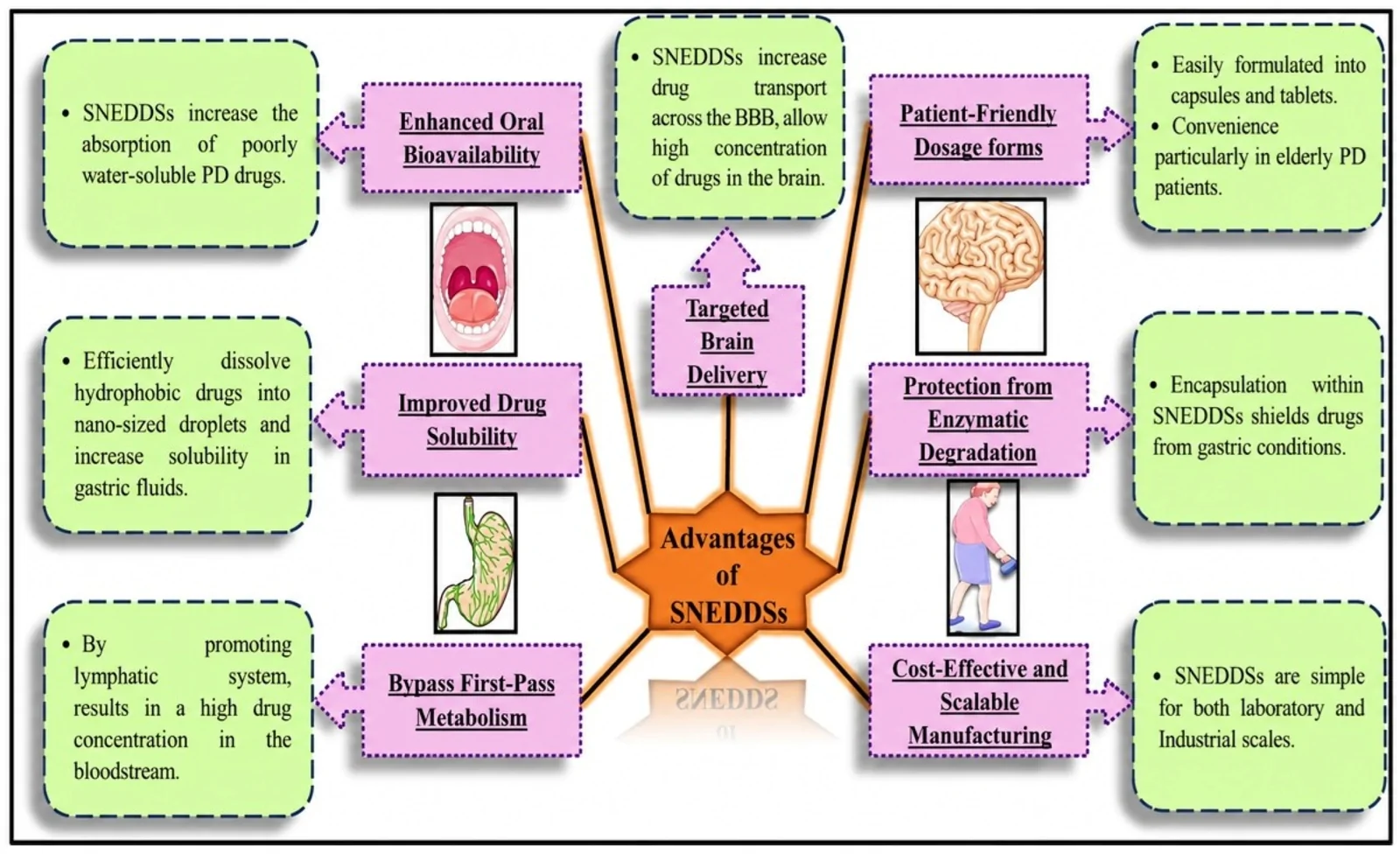
Major advantages of SNEDDS therapy for PD.

In addition, a higher plasma concentration obtained through SNEDDS delivery produces a powerful concentration gradient through the BBB, favoring drug diffusion into the brain. The mechanism of brain-targeted drug delivery by SNEDDSs via the lymphatic route is depicted in Figure 2. It includes (Figure 2A) oral administration and epithelial absorption, (Figure 2B) chylomicron-mediated transport and lipid digestion, and (Figure 2C) systemic circulation that avoids first-pass metabolism for improved brain delivery. SNEDDSs’ small size enables greater paracellular transport and endocytosis by BBB endothelial cells to facilitate drug penetration more effectively than larger particles. Some SNEDDS formulations also include surfactants (Tween 80 or the polysorbates family) or targeting ligands, which can modulate BBB tight junctions or engage in receptor-mediated transcytosis to further increase brain uptake [111,112,113]. Besides enhancing bioavailability and brain targeting, SNEDDSs also prevent enzymatic degradation of drugs in the gut, maintain stability, and enable controlled release, which is particularly beneficial for sensitive molecules like antioxidants, e.g., curcumin and resveratrol [114]. With these multifaceted benefits—solubilization enhancement, metabolic protection, nanoscale transport facilitation, and BBB interaction—SNEDDSs present a revolutionary platform for enhancing the therapeutic effectiveness of orally administered CNS-targeted drugs over other nanocarrier systems. They represent a significant step forward in patient-friendly neurotherapeutics, both delivering better drug performance and better quality of life for patients suffering from PD (Figure 3) [115]. When assessed together, SNEDDSs appear to provide an appealing patient-friendly nanocarrier delivery mechanism for the treatment of PD. However, most of the existing knowledge in this area remains mostly theoretical and preclinical. Some of the key weaknesses in this respect include questions relating to the safety of the excipients when used continuously, the consistency of results in different lipid-based systems, and the complete lack of clinical trials dedicated to the condition under consideration.

**Table 3:** Outline of nanotechnology-driven strategies for PD: types, advantages, and challenges.

Types of Nanoformulation	Advantages	Challenges	Why SNEDDSs	Reference
Polymeric nanoparticles (PLGA, chitosan, etc.)	Enhance drug stability, penetrate the BBB	Possible toxicity and challenges in scale-up	SNEDDSs nullify polymer toxicity and simplify for scalability	[116,117]
Liposomes/nanogels/solid lipid nanoparticles	Increase drug loading, biocompatible, improved BBB penetration	Shorter half-life, costly	SNEDDSs offer a longer shelf life and are cost-effective	[116,118]
Metallic nanoparticles	Facilitate dual functionality in therapy, precise delivery, and enhanced diagnosis	Potential neurotoxicity and unclear long-term compatibility	SNEDDSs avoid metal-based toxicity	[119,120]
Carbon-based nanoparticles (carbon dots, nanotubes)	Increase surface area, an effective vehicle for drug	Issues about biodegradability, long-term safety, and regulatory challenges	SNEDDSs use biodegradable lipids, bypassing the hurdle of carbon nanomaterials	[120,121]
Scaffold/hydrogel-based nanotechnology	Facilitates cell and gene transplantation.	Invasive intervention, stability	SNEDDSs are non-invasive and offer better stability	[122,123,124]

## 5. Mechanistic Rationale for SNEDDSs in PD

PD essentially results from oxidative stress, neuroinflammation, mitochondrial dysfunction, impaired protein clearance, and progressive BBB disruption. All these factors render conventional pharmacotherapies less effective. These disease-specific obstacles result in poor solubility, extensive first-pass metabolism, fluctuating plasma levels, and inconsistent CNS exposure of drugs administered orally. While SNEDDSs can improve solubility, dissolution, and intestinal absorption of poorly water-soluble Parkinson’s drugs, such outcomes still need to be justified through in vitro and in vivo studies [125,126]. The use of lipidic excipients for the incorporation of therapeutic agents facilitates their uptake via the lymphatic system, thus partially bypassing hepatic first-pass metabolism and resulting in higher systemic availability. Most importantly, SNEDDSs may increase systemic exposure of dopaminergic drugs, antioxidants, and anti-inflammatory agents, thus enabling combined modulation of oxidative stress and neuro-inflammatory pathways. Greater systemic exposure with SNEDDSs does not automatically mean effective delivery across the BBB. To confirm whether these formulations truly raise drug levels at relevant CNS sites, dedicated brain-specific pharmacokinetic studies are still required.

SNEDDSs provide formulation versatility and patient-related advantages. These systems, composed of oils, surfactants, and cosurfactants, spontaneously nanoemulsify in the gastrointestinal tract, thereby improving the oral bioavailability of poorly water-soluble drugs, a major limitation in PD therapy. Their adaptability allows easy incorporation into capsules or tablets, ensuring cost-effective, reproducible, and stable formulations suitable for elderly patients who require simplified dosing regimens [127]. By enhancing drug transport across biological barriers and potentially increasing brain exposure, SNEDDSs extend therapeutic reach while maintaining practicality in clinical use. The growing interest in SNEDDSs is highlighted by successful commercial formulations such as Gengraf^®^ by AbbVie Inc., Neoral^®^ by Novartis, Fortovase^®^ by Roche Laboratories Inc., and Agenerase^®^ by GlaxoSmithKline, which exemplify SNEDDSs’ translational potential [128]. Collectively, SNEDDSs represent not just a passive delivery vehicle but a strategic platform that complements the multifactorial pathophysiology of PD, positioning them as a promising approach to improve treatment efficacy and accessibility.

## 6. Formulation Design and Therapeutic Applications of SNEDDSs for Parkinson’s Disease

Prior to exploring SNEDDSs, it is necessary to point out that every single ingredient utilized in PD therapy is different and has a very important function in the drug delivery system. SNEDDS effectiveness largely relies on the appropriate choice and mix of oils, surfactants, and cosurfactants [127,128]. These components are planned to allow the economical encapsulation, protection, and release of a drug, especially poorly water-soluble ones, which is the main problem in the pharmacological fight against PD. With increased uptake not only through the gastrointestinal route but also through the lymphatic route, SNEDDSs thus facilitate the transport of drugs to the brain across the BBB and help overcome the key barriers of solubility and metabolism during the first pass [128]. The following sections of this article explicate the main role of each part in the realization of SNEDDS-based therapies for PD.

### 6.1. Oil Phase

Oils are an essential component of SNEDDSs since they have the ability to solubilize a lipophilic drug as well as to increase the drug’s absorption from the GI tract by facilitating its passage through the intestinal lymphatic system, thus enhancing permeability across the BBB to target PD [129]. Nanoformulations based on lipids improve drug transport to the brain by increasing penetration through the BBB, providing selective treatment, and minimizing side effects. Their potential for carrying anti-Parkinson drugs makes them ideal transfer vehicles for PD with the most effective treatment. Nanoparticles are one of the main ways that these are transported across the BBB; this happens when they adsorb apolipoproteins like apolipoprotein E (ApoE), which thus allows them to go through receptor-mediated transcytosis via the low-density lipoprotein (LDL) that is found on brain capillary endothelial cells [130]. Further, lipid-based systems also deliver several advantages, including higher drug loading capacity, better encapsulation of lipophilic drugs, and improved stability during storage. Such qualities not only improve drug bioavailability and brain targeting but also facilitate the long-term stability and efficiency for PD [131]. The choice of oil phase plays a critical role in enhancing drug solubility and absorption; long-chain triglycerides (LCTs) and medium-chain triglycerides (MCTs) are commonly used due to their ability to promote lymphatic transport and improve the bioavailability of hydrophobic drugs. Moreover, oils like peppermint oil, clove oil, and eucalyptus oil have been used in SNEDDS formulations not just because of their solubilizing ability but also because of their intrinsic antioxidant, anti-inflammatory, and neuroprotective properties, which may add to the therapeutic efficacy of managing PD [132]. Furthermore, if oils are utilized along with a suitable surfactant that enhances their solubility, the oils will improve emulsification properties as well as increase drug loading capacities. These novel lipidic materials have the capacity to mimic surfactants and are hence amphiphilic [133]. Oils are thus a crucial component of SNEDDSs as they not only act as vehicles for lipophilic drugs but also improve solubility, encourage circulation of lymphatics, and greatly increase drug absorption [104].

### 6.2. Surfactants

Surfactants participate in lowering interfacial tension between water and oil phases, as well as in the process of stabilizing emulsions in SNEDDSs. Surfactants thus play roles as emulsifiers, wherein they assist with droplet size regulation, drug release rate, and system stability [134]. Surfactants further utilize extracellular membrane signaling for inhibiting the biotransformation functions of CYP3A4 [135]. In addition, by hindering the ATP-binding cassette, non-ionic surfactants are also P-glycoprotein efflux inhibitors. Additionally, by altering the gastrointestinal lumen’s lateral membrane packing and facilitating passive transport across the gut wall, non-ionic surfactants increase drug absorption [136]. Usually, surfactants are grouped based on their hydrophilic–lipophilic balance (HLB value) and charge. Three important factors should be considered when selecting a surfactant for SNEDDSs: the ability of the surfactant to emulsify, the HLB value of the surfactant, and the extreme solubility of the drug. The size of emulsion particles can be reduced by adding more surfactant since it reduces surface tension at the interface of oil and water, which lowers the free energy for emulsification [137]. However, due to excessive water penetration in lipid droplets, which causes significant disturbance of the oil–water interface and relaxation of highly polydisperse nanoemulsion droplets, an increase in surfactant quantity may occasionally result in larger particle sizes [138,139].

They are the main building blocks for constructing nanocarrier-based drug delivery systems because of their properties that improve solubility, stabilize formulations, and help transport drugs across the BBB for PD treatment [12]. The most important amongst them is Polysorbate 80 (PS 80)—a non-ionic surfactant from the Tween family—which has unique properties that promote brain-targeted delivery by allowing BBB crossing, which occurs due to the facilitated adsorption of plasma apolipoproteins, in particular, the one that is called ApoE, onto the nanoparticle surface [140]. Additionally, PS 80 is known to inhibit P-glycoprotein (P-gp) and other ATP-binding cassette (ABC) efflux transporters, helping increase drug retention in the brain by reducing premature drug efflux. These properties are particularly crucial for treating disorders of ND, where efficient brain penetration is essential [141]. Moreover, the regulatory status of the surfactant (e.g., GRAS—generally considered safe) and its appropriateness for oral delivery should also be considered when choosing a surfactant [111]. After oral administration, some SNEDDS formulations may irritate the GI epithelium. To minimize irritation, it is important to keep the surfactant content of SNEDDSs as low as feasible.

### 6.3. Cosurfactants

Low interfacial tension, required for spontaneous formation of stable nanoemulsions within SNEDDSs, is rarely achieved by a single surfactant. Therefore, a cosurfactant or cosolvent has to be added to enhance emulsification efficiency and formulation performance [142]. Cosurfactants synergistically combine with surfactants, reduce interfacial tension significantly, and enhance the flexibility of the oil–aqueous phase interfacial film. This allows for rapid emulsification of fine and thermodynamically stable nanoemulsions upon dilution with gastrointestinal fluids [143,144]. In addition, cosurfactants also assist in enhancing solubilization of sparingly water-soluble drugs in the oil phase and surfactant dispersibility, thereby generally boosting the stability, uniformity, and homogeneity of the nanoemulsion. Cosurfactants also reduce the risk of precipitation of the drug and enhance uniform dispersion of the drug [145]. Typically used cosurfactants and cosolvents are low-molecular-weight, amphiphilic compounds like propylene glycol, ethanol, polyethylene glycol (PEG), and, more recently, Transcutol^®^ HP, which have been shown to be effective in optimizing SNEDDS performance for the treatment of PD [146]. Not only does their addition improve thermodynamic stability, but it also leads to reproducible drug absorption profiles; hence, they are a vital element in the successful optimization and formulation of SNEDDSs.

The selection of excipients is also a critical translational consideration for SNEDDSs intended for chronic oral administration in ND. Commonly employed components include medium-chain triglyceride derivatives (e.g., Capryol^®^ 90), polysorbates such as Tween 80, propylene glycol, polyethylene glycols, and lipidic surfactants, which have established histories of oral acceptability and are listed as GRAS or FDA-approved excipients [147]. McCartney and coworkers demonstrated that Capryol^®^ 90 at concentrations up to 40 mg/mL produced no overt mucosal toxicity in rat intestinal tissues, while Hamid reported that propylene glycol (≤10% *v*/*v*), Tween 80 (10% *v*/*v*), and PEG 400 (≤10% *v*/*v*) were safe without significant intestinal damage. In contrast, Gelucire^®^ 44/14 and Cremophor EL at 10% showed mild increases in toxicity markers, suggesting caution at higher concentrations [148,149]. These findings confirm that excipients widely used in SNEDDSs are safe within established GRAS/FDA ranges for oral delivery.

**Figure 4:**
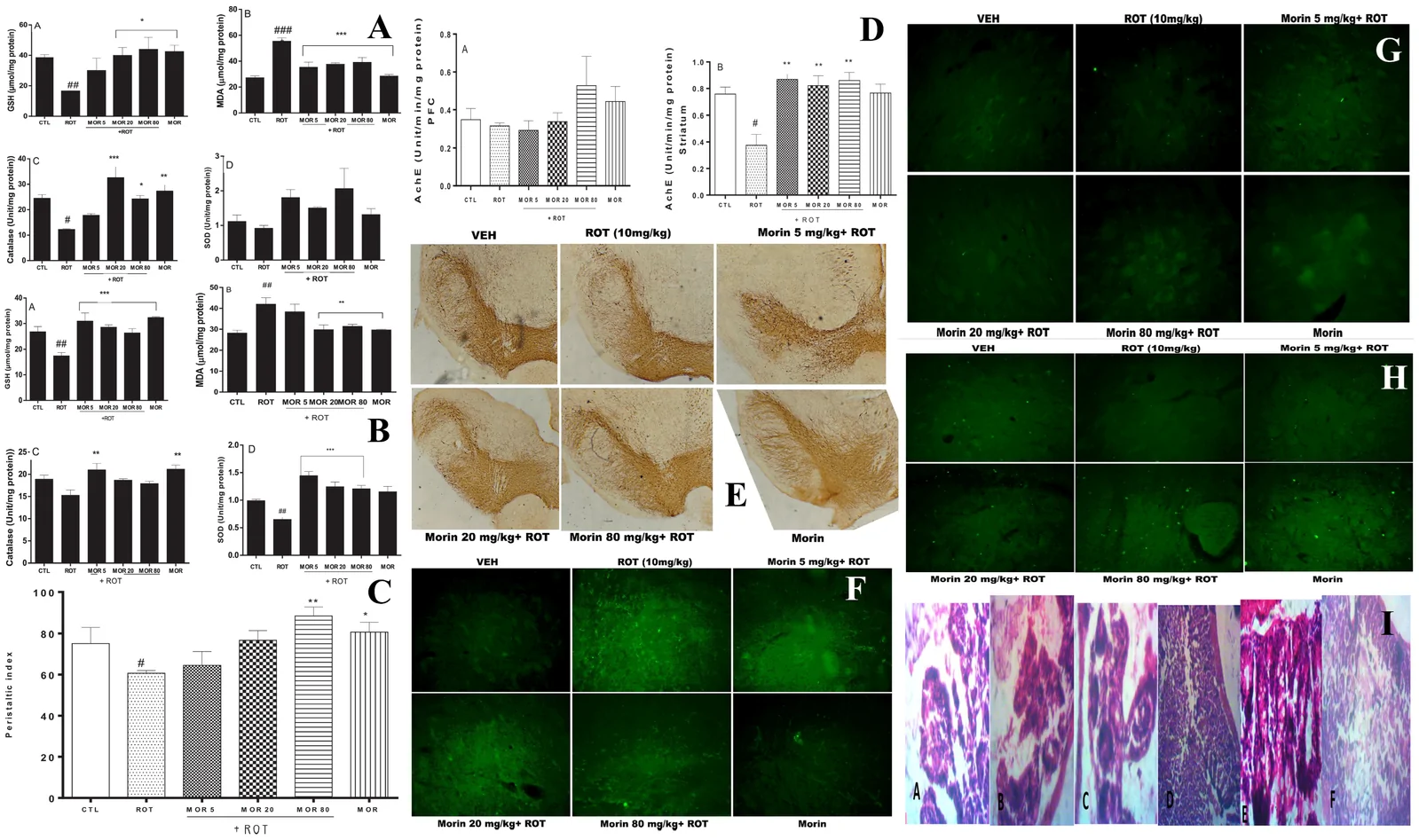
Study showing potential morin-loaded SNEDDSs against PD in a male albino mouse model. Morin-loaded SNEDDSs exhibited an increase in GSH, catalase, and SOD and a decrease in MDA levels, similar to that of the negative control group, ^#^ *p* < 0.05, ^##^ *p* < 0.01, ^###^ *p* < 0.001 versus vehicle-control treated, * *p* < 0.05, ** *p* < 0.01, *** *p* < 0.001 versus vehicle-rotenone treated (**A**). In the striatum, morin-loaded SNEDDSs exhibited an increase in GSH, catalase, and SOD and a decrease in MDA levels, ^##^ *p* < 0.01 versus vehicle-control treated, ** *p* < 0.01, *** *p* < 0.001 versus vehicle-control treated group (**B**). SNEDDSs increased the peristaltic index in intestinal motility, ^#^ *p* < 0.05 versus vehicle control treated group, * *p* < 0.05, ** *p* < 0.01 versus rotenone treated group (**C**). When rotenone was administered orally, it decreased acetylcholinesterase activity in the striatum. Effect of treatments on cholinergic activity in the striatum, ^#^ *p* < 0.05 versus vehicle control treated, ** *p* < 0.01 versus vehicle-rotenone treated (**D**). Decrease in TH (**E**). Similarly, SNEDDSs exhibited a protective and supportive glial response, which is required in mitigating PD (**F**). Reduced iba-1 immunoreactivity is shown in (**G**). Morin-loaded SNEDDSs attenuated the rotenone-induced upregulation of TLR-4 expression in the striatum (**H**). In histological assessment, morin effectively protected against rotenone-induced colonic epithelial necrosis, effect of morin and rotenone treatments on the histoarchitecture of mouse colon (A) vehicle control (B) ROT (10 mg/kg) (C) morin 5 mg/kg + ROT (D) morin 20 mg/kg + ROT (E) morin 80 mg/kg + ROT and (F) Morin 20 mg/kg only (**I**). License Number 6176390987329 [150].

Choosing and optimizing the oil, surfactant, and cosurfactant are very crucial in enhancing the effectiveness of SNEDDSs to the maximum extent, as they determine the solubilization of the drug, the droplet size, and the absorption profile. If an SNEDDS is well designed, it can significantly enhance the transport of poorly soluble drugs. Most recently, it has been confirmed by various studies that the potential benefit of SNEDDSs lies in the improvement in the pharmacokinetic properties and therapy of different anti-Parkinsonian drugs. Garg et al. formulated a paroxetine SNEDDS, consisting of Caproyl 90, Cremophor EL, and propylene glycol—which resulted in a 2.3-fold rise in a rat model of depression and significantly ameliorated serotonergic and noradrenergic activity. The results of this study indicate that they not only increased systemic exposure but also potentiated CNS efficacy [112]. On the other hand, a ropinirole-loaded SNEDDS formulated by Detholia et al. showed the possibility of an optimized formulation, droplet size (~96.7 nm), rapid emulsification (22 s), and a 3.3-fold increase in drug release, thus making it suitable for further in vivo studies [113]. Besides that, natural bioactives such as morin and α-pinene showed outstanding treatment results when they were carried through SNEDDSs. For example, Ishola et al. studied the potential of morin-loaded SNEDDSs against PD in a male albino mouse model. The results of the study demonstrated that morin-loaded SNEDDSs exhibited a 2.22-fold, 2.07-fold, and 1.51-fold increase in GSH, catalase, and SOD and a 1.65-fold decrease in MDA levels compared to the negative control group (Figure 4A). In the striatum, morin-loaded SNEDDSs exhibited 1.83-fold, 0.68-fold, and 0.58-fold increases in GSH, catalase, and SOD and a 1.5-fold decrease in MDA levels compared to the negative control group (Figure 4B). The effect of morin-loaded SNEDDSs on the peristaltic index in intestinal motility showed a 1.3-fold increase compared to the rotenone-induced negative control group (Figure 4C). When rotenone was administered orally, it caused a significant decrease in acetylcholinesterase activity in the striatum when compared with a vehicle-treated control. One-way ANOVA revealed a significant effect of treatments on cholinergic activity in the striatum by 2.3-fold but not in the prefrontal cortex (Figure 4D). Morin-loaded SNEDDSs ameliorated the decrease in TH (tyrosine hydroxylase)-positive neurons, which is caused by rotenone (Figure 4E). Similarly, the morin-loaded SNEDDS-treated group exhibited an increase in GFAP cells compared to other groups. This exhibited a protective and supportive glial response, which is required in mitigating PD (Figure 4F). In another experiment, morin administration significantly reduced iba-1 immunoreactivity when compared with the vehicle–rotenone-treated group (Figure 4G). Investigating the neuroprotective mechanisms of morin, its administration significantly attenuated the rotenone-induced upregulation of TLR-4 expression in the striatum (Figure 4H). In histological assessment, morin at 20 mg/kg effectively protected against rotenone-induced colonic epithelial necrosis, unlike 5 or 80 mg/kg doses (Figure 4I). The formulation attenuated oxidative stress, as evidenced by reduced lipid peroxidation and restoration of endogenous antioxidant defenses, including glutathione (GSH), catalase, and superoxide dismutase (SOD). It also reduced neuro-inflammatory responses, as indicated by decreased expression of microglial marker Iba-1, astrocytic marker GFAP, and Toll-like receptor 4 (TLR4). Furthermore, treatment preserved tyrosine hydroxylase (TH) expression in the substantia nigra, suggesting protection of dopaminergic neuronal integrity. Their study strongly indicated the multi-neuroprotective potential of morin [150]. Similarly, α-pinene packed into an SNEDDS, which was formulated by Srivastava, showed promising results, including increased Sh-SY5Y cell viability, improved motor behavior, and increased antioxidant enzyme levels. The pharmacological role of the ALP-SENF was validated through both in vitro and in vivo experimental models of Parkinson’s disease. In SH-SY5Y cells challenged with 6-OHDA, ALP-SENF at 100–200 µM significantly improved neuronal viability, demonstrating a neuroprotective effect comparable to the antioxidant reference N-acetyl-L-cysteine. In vivo, ALP-SENF produced pronounced improvements in motor performance and behavioral outcomes in reserpine antagonism and haloperidol-induced Parkinsonism models, outperforming the plain α-pinene suspension. Biochemical assays further revealed a marked enhancement in endogenous antioxidant enzymes, indicating that the formulation mitigates oxidative stress-induced neurodegeneration primarily through free radical scavenging and reinforcement of antioxidant defense pathways. Collectively, these findings establish ALP-SENF as a promising pharmacological candidate for PDfunctioning as both an antioxidant and neuroprotective agent that preserves neuronal integrity and improves motor outcomes [151]. The incorporation of a *Citrus trifoliata* extract (hesperidin) within a SNEDDS matrix induced a drastic reduction in the MAO-B inhibitory concentration (IC_50_ decreased to 129.9 ng/mL from 707.7 ng/mL with pure hesperidin), thus demonstrating the formulation’s role in facilitating enzymatic inhibition and ability to exert anti-Parkinson activity at lower doses [152]. In the same manner, a fisetin SNEDDS also provided 3.79 times higher permeation, and antioxidant enzymes (SOD, catalase, GSH) were considerably upregulated and neuro-inflammatory cytokines (TNF-α, IL-6) were suppressed. The ELISA experiments further revealed the intervention of BDNF and α-synuclein, which are main players [153]. Hussain et al. (2018) chose a BCS class II drug, bromocriptine mesylate, for SNEDDS formulation with enhanced dissolution and absorption, which aids in enhancing oral bioavailability [154]. Vadlamudi and coworkers concluded that an entacapone S-SMEDDS with optimized formulation exhibited rapid and high drug release (~91% within 60 min), along with improved physicochemical stability and shelf life. These improvements were associated with a stronger anti-Parkinsonian response in the chlorpromazine-induced model, as indicated by a reduction in catalepsy. At the biochemical level, the S-SMEDDS attenuated oxidative and nitrosative stress-associated abnormalities: the optimized AG8 formulation reduced TBARS from 34.25 to 27.82 nmol/L/mg protein and nitrite from 10.53 to 6.28 nmol/L/mg protein relative to pure entacapone while increasing reduced glutathione (GSH) from 14.98 to 20.74 nmol/L/mg protein and catalase activity from 114.94 to 126.85 mmol/L/mg protein, respectively [155]. Sharma et al. (2016) also formulated a rutin-loaded SNEDDS with deep antioxidant potency and neuroprotection in rotenone-induced models of PD [156]. Pharmacokinetic examination also revealed a 2.3-fold enhanced oral bioavailability, which demonstrates its efficiency for application in the therapeutic management of oxidative stress in PD [156]. Coformulations such as vitamin E and rutin SNEDDSs not only improved drug solubility and systemic availability (~2.3-fold) but also brought about a strong antioxidant effect and behavioral improvement in haloperidol-induced PD models, thus indicating a synergistic effect of neuroprotection [156]. In the same way, coenzyme Q10 SNEDDSs were far superior to the traditional ones in their capability of increasing AUC by almost five times, improving C_max_, and shortening T_max_, thus confirming that they can supply therapeutic concentrations more quickly and for a longer duration [157]. SNEDDS research has mainly been focused on finding ways to improve oral delivery of drugs with poor bioavailability by increasing drug solubilization and absorption.

## 7. Probiotic-Integrated SNEDDSs for Gut–Brain Axis Modulation in Parkinson’s Disease

Probiotics are live microorganisms that, when administered in adequate amounts, confer health benefits to the host. Their importance lies in their ability to restore gut microbial balance, strengthen intestinal barrier function, modulate immune responses, and influence systemic physiology through the gut–brain axis. In recent years, probiotics have been increasingly applied to manage metabolic disorders, inflammatory bowel disease, and neurodegenerative conditions, owing to their capacity to reduce oxidative stress, suppress pro-inflammatory cytokines, and regulate neurotransmitter synthesis [158]. This makes them particularly relevant in PD, where gut dysbiosis, intestinal permeability, and chronic inflammation are recognized contributors to disease progression.

**Figure 5:**
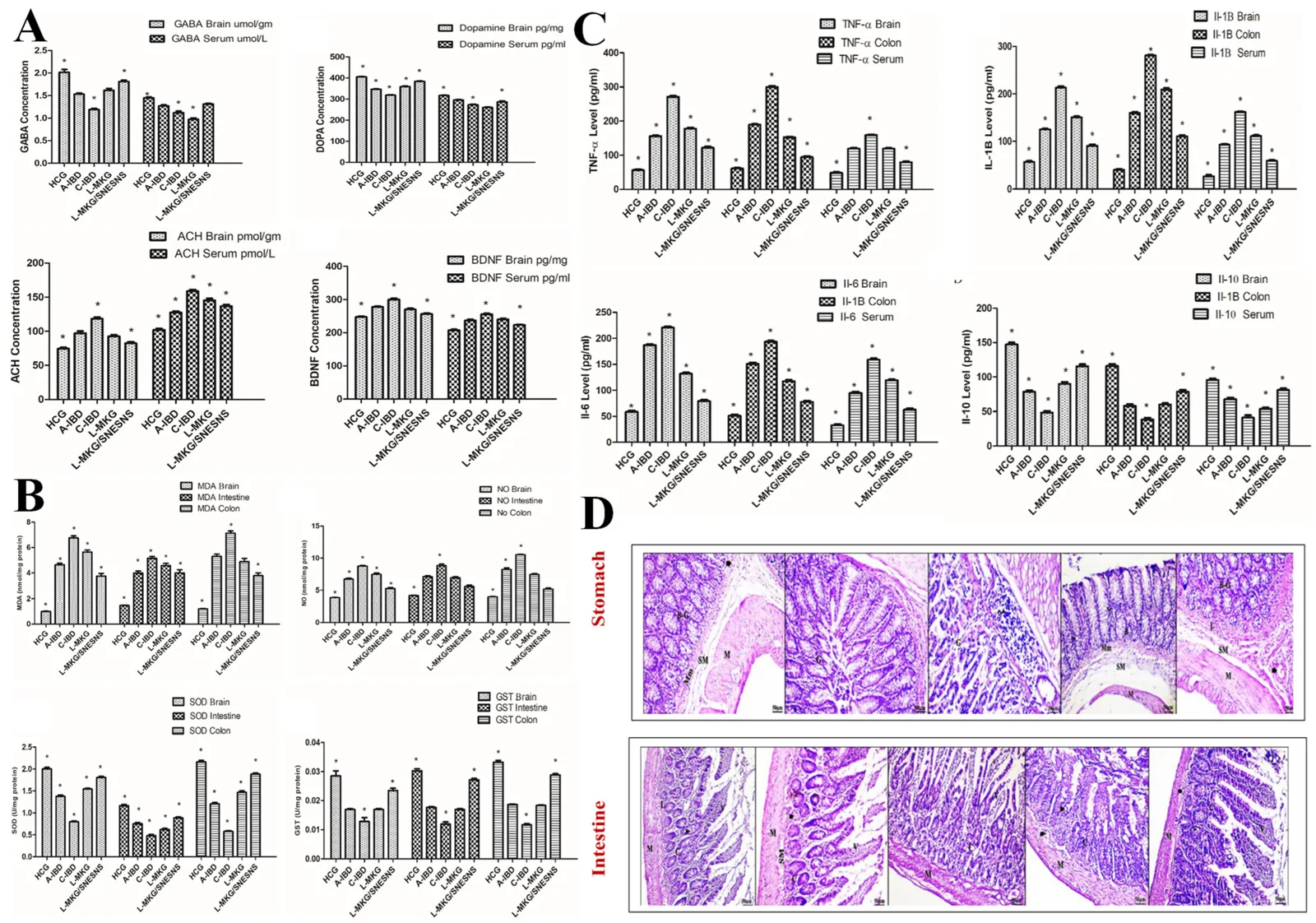
Study showing the effects of milk kefir-based SNEDDSs on cognitive enhancement via the microbiota–gut–brain axis. Groups marked with the same asterisk indicate significance (* *p* < 0.05), in (**A**) inflammatory cell activation, (**B**) triggered LPS serum levels, pro-inflammatory cytokines, and intestinal permeability in an IBD-DSS-induced rat model, and (**C**) probiotics. (**D**) Improvement in previously damaged intestinal and stomach tissues. Reproduced from Anwar et al. [160], © 2024 by the authors, licensed under Creative Commons Attribution 4.0 International (CC BY 4.0).

Probiotic-loaded SNEDDSs have been able to achieve a level of therapy over conventional probiotics, especially in gut microbiota modulation, suppression of inflammation and oxidative stress, and re-establishment of gut–brain axis balance. This dual-function strategy positions probiotic-assisted SNEDDSs as a novel and transformative oral delivery platform, warranting focused investigation in neurodegenerative and inflammation-driven disorders and offering a compelling direction for future high-impact research. Khursheed et al. developed an S-SNEDDS of curcumin using a unique combination of *Ganoderma lucidum* extract powder and probiotics as multifunctional solid carriers. The optimized spray-dried spheroidal S-SNEDDS exhibited nano-sized droplets (35–37 nm), high drug loading (95–96%), favorable zeta potential (−21.48 to −23.22 mV), and rapid drug release exceeding 90% upon reconstitution. Importantly, the formulation significantly enhanced curcumin permeability across Caco-2 cell monolayers (*p* < 0.05) while maintaining comparable physicochemical and dissolution characteristics to liquid SNEDDSs, indicating excellent formulation stability [159]. Supporting this, Anwar M.M. et al. conducted a study to check the impact of lyophilized milk kefir-based SNEDDSs on cognitive enhancement via the microbiota–gut–brain axis, and they reported various studies like (Figure 5A) 5-HT for direct inflammatory cell activation, in which GABA concentration increased by 1.5-fold and DOPA concentration increased by 1.11-fold, while ACH and BDNF concentrations decreased by 1.07-fold and 1.14-fold simultaneously. For further justification, they also (Figure 5B) triggered LPS serum levels, pro-inflammatory cytokines, and intestinal permeability in an IBD-DSS-induced rat model, which showed that TNF-α, IL-1β, and IL-6 decreased by 1.75-fold, 2.16-fold, and 2.07-fold, simultaneously, and IL-10 increased by 0.625. When antioxidants were administered in the brain, colon, and intestine (Figure 5C) individually, SNEDDSs loaded with probiotics showed better results. In microscopic examination of intestinal and stomach sections (Figure 5D), L-MKG/SNESNS resulted in significant improvements in the previously damaged intestinal and stomach tissues when administered daily. All these results show that lyophilized probiotics co-loaded as S-SNEDDSs enable selective targeting of inflamed intestinal mucosa. This strategy alleviated spatial memory impairment, promoted intestinal healing responses, and revealed gut-driven neurobiological alterations within the central nervous system in an IBD-like model [160]. Notably, this probiotic-assisted SNEDDS approach demonstrated superior efficacy over probiotics alone in regulating neurotransmitter levels and improving cognitive functions, highlighting its potential to address gut–brain axis-associated disorders through combined probiotic and SNEDDSs.

The available studies, when combined, pointed to SNEDDSs as a clever and very effective oral delivery platform capable of helping therapeutic compounds bypass solubility, permeability, and stability issues. Furthermore, SNEDDSs function as multifunctional systems that not only contain a synthetic drug but can also hold a combination of phytochemicals and probiotics, thus enabling a synergistic control of oxidative stress, inflammation, intestinal integrity, gut microbiota, and gut–brain axis signaling. Although there have been quite a few exciting results, probiotic-carrying and bioactive-loaded SNEDDSs are still, to a great extent, an untouched area in the case of PD. Research in the future needs to be mechanistic in nature, validated by long-term in vivo studies, so that the complete double therapeutic and delivery potential of SNEDDSs is realized, allowing them to become the new generation of oral platforms for complex and multifactorial diseases. A detailed list of applications of the SNEDDS formulation comprising Parkinson’s drugs and probiotics is given in Table 4.

**Table 4:** Application of SNEDDSs for PD.

Therapeutic Moiety	Key Component	Inducing Agent/Model/Study	Results	References
Paroxetine	Caproyl 90, Cremophore EL, and propylene glycol	Depression model rats	Characterization of the optimized batch showed results in the range. Improved behavioral activity by decreasing the depletion of serotonin and norepinephrine. Oral bioavailability increased by 2.3 times in comparison to the suspension.	[112]
Ropinirole	Capmul MCM EP, Tween 20, Aerysol EL 135, Syloid 244 FP	In vitro	Optimized using the BBB. Droplet size was 96.71 nm; emulsification time was 22 s for optimized SNEDDSs. SNEDDSs improved release by 3.3-fold compared to pure ropinirole.	[113]
Morin (3,5,7,2′,4′-pentahydroxyflavone)	-	Rotenone-induced PD mouse model	Reversed depression behavior and MDA. Restored GSH, SOD, AChE Suppressed α-synuclein, GFAP, Iba-1, TLR-4.	[150]
α-pinene	Anise oil, Tween 80, Transcutol HP	Pilocarpine antagonism rodent model	Characterization of the optimized batch showed results in the range. Tremulous jaw movements, body temperature, salivation, and lacrimation showed improved results in comparison with a plain suspension.	[151]
Citrus trifoliata extract (Hesperidin)	Isopropyl Myristate, Tween 80, and PEG 200,	MAO-B enzyme assay, and molecular docking	Decreased IC_50_ (129.9 ng/mL) vs. an extract (252.7) and pure hesperidin (707.7). SNEDDSs boosted MAO-B inhibition.	[152]
Fisetin	Castor oil, Lauroglycol FCC, Tween 80, Transcutol P	Rotenone-induced female Sprague Dawley rats	A 3.79-fold increase in permeation. Increased levels of SOD, catalase, and GSH. Decreased TNF-α and IL-6. ELISA confirmed a decrease in α-synuclein and an increase in BDNF levels.	[153]
Bromocriptine mesylate	Castor oil, Tween 80, ethanol	In vitro	Oral bioavailability increased by 3.29-fold with pure BM powder. Showed a promising delivery system to improve solubility, dissolution, and stability.	[154]
Entacapone	Gingelly oil, rice bran oil, Span 20, Tween 80, glycerin, propylene glycol	CPZ-induced PD model	S-SMEDDSs showed a 2.12-fold increase in drug release compared to the suspension. G8 showed a decrease in catalepsy score, TBARS, and nitrate level, with augmented glutathione and catalase levels.	[155]
Vitamin E and rutin	Sefsol 218 + vitamin E (1:1), SolutolHS15 and Transcutol P	Haloperidol-induced PD rat model	SNEDDSs improved bioavailability by 2.3-fold from rutin suspension. Higher locomotory activity, better muscle coordination, shorter cataleptic duration, and the akinesia test showed that movement improved. Increased GSH/SOD. Decreased TBARS. Enhanced antioxidant effect.	[156]
Coenzyme Q10	Labrasol, Transcutol P, Lauroglycol FCC	Wistar rats (pharmacokinetic)	S-SMEDDSs showed a 1.69-fold increase in drug release from the market formulation. AUC increased by 5 times, Tmax decreased, and Cmax increased vs. Co-Q10 powder.	[157]
Curcumin	Ganoderma lucidum extract powder + probiotics	Caco-2 permeability study	Droplet size 35–37 nm; drug loading 95–96%; zeta potential −21.48 to −23.22 mV; >90% drug release. Enhanced Caco-2 permeability (*p* < 0.05).	[159]
Licorice extract	Lyophilized milk kefir	DSS-induced IBD	↓ TNF-α (1.75-fold), IL-1β (2.16-fold) and IL-6 (2.07-fold); ↑ IL-10 (0.625-fold). Improved oxidative stress parameters, intestinal/gastric histology, neurochemical alterations and spatial memory.	[160]

## 8. Pathogenesis-Driven Drug Repurposing for PD

Current treatments for Parkinson’s disease are symptomatic in nature and focus on motor improvement without changing the course of the disease. A practical approach to enhancing the development of new treatments is by using existing drugs with a proven safety profile, pharmacokinetic information, and an approved status. This helps reduce the cost and time needed to bring a treatment from the bench to the clinic when compared to new drug development. One issue that needs to be considered in the process of developing new medications is that a number of molecules that show promise for PD are not able to move forward due to issues like poor solubility and fast clearance.

Repurposed drugs can be carefully matched to the pathophysiology of PD. For instance, baicalein, gallic acid, and ginsenosides tackle oxidative stress and neuroinflammation; rifampicin and cuminaldehyde are active against α-synuclein aggregation; ambroxol serves as a chaperone in lysosomes that can reverse glucocerebrosidase function in GBA-linked PD; and safinamide offers dual dopaminergic and non-dopaminergic effects. All these examples of repurposed compounds demonstrate the possibility of affecting pathophysiological processes such as mitochondrial dysfunction, protein aggregation, and gut microbiome alterations. Nevertheless, their medicinal effect is limited by the biopharmaceutical properties of repurposed drugs, thus making SNEDDSs an attractive delivery system.

SNEDDSs have proven to be more relevant for the repurposing of BCS II and IV compounds, which constitute the majority of candidates for drug repurposing in PD. Through increased solubilization, intestinal absorption, and lymphatic transport, SNEDDSs may increase the systemic bioavailability and CNS penetration of these drug classes. In addition to small molecules, probiotics may serve as an alternative source of repurposing, with strains like *Lactobacillus reuteri* and *Bifidobacterium longum* demonstrating neuroprotective effects by acting on the gut–brain axis. The already existing application of SNEDDSs in other indications (antifungals, immunosuppressants) provides an additional reason to explore their potential in PD.

Although there is clear rationale for the use of repurposed drugs in PD, much of the evidence for their use is preclinical. At this time, no SNEDDS formulations have been tested in clinical trials specifically for PD patients. There is a need for a long-term safety assessment of the excipients for chronic use in neurodegenerative disorders since tolerance may differ depending on the type of lipids used. Issues related to food effect, variability in droplet size, and reproducibility across batches are other factors that need to be considered.

From the perspective of repurposing, various pharmaceutical molecules with a good safety profile and different therapeutic applications have shown specific features that match the disease mechanisms involved in PD. Such molecules, which were previously developed for treating infectious diseases, metabolic diseases, or as phytochemicals from natural products, possess antioxidant, anti-inflammatory, antiaggregation, and mitochondria-protective effects; thus, they are considered potential candidates for repurposing in PD treatment. The significance of these agents is their involvement in mechanisms such as oxidative stress, α-synuclein aggregation, mitochondrial dysfunction, and neuroinflammation, all of which are crucial in PD pathology.

Baicalein has emerged as a prototypical pathogenesis-driven repurposing candidate in PD due to its combined antioxidant, anti-inflammatory, and α-synuclein-modulating activities. Extensive preclinical evidence demonstrates that baicalein directly interferes with α-synuclein oligomerization and fibrillation while simultaneously attenuating oxidative stress and inflammatory signaling, thereby protecting dopaminergic neurons from aggregation-induced toxicity [161]. Similarly, gallic acid, a potent polyphenolic antioxidant and anti-inflammatory molecule, has shown significant improvements in penetration, sustained release, and reduced mucosal irritation when formulated as a self-nanoemulsifying system in non-neurological applications. Given the central role of oxidative stress and neuroinflammation in PD pathogenesis, gallic acid represents a compelling formulation-limited candidate whose neuroprotective potential may be substantially enhanced through SNEDDS-based delivery [162]. Ginsenosides, particularly Rg1, Rb1, and rare ginsenoside derivatives, further exemplify multi-target neuroprotective agents, exerting disease-modifying effects through attenuation of neuroinflammation, suppression of oxidative stress, inhibition of apoptosis, restoration of mitochondrial function, and regulation of the PI3K/Akt, BDNF/TrkB, NF-κB, Nrf2, and Wnt/β-catenin signaling pathways, yet remain constrained by poor oral bioavailability [163].

Several clinically approved or advanced investigational agents have also demonstrated strong mechanistic relevance to PD but remain underutilized due to pharmacokinetic limitations. Rifampicin, a widely used antibiotic, exhibits potent antiaggregative activity against α-synuclein by inhibiting fibril formation, disaggregating preformed fibrils, and reducing phosphorylated α-synuclein levels in transgenic models of synucleinopathies [164,165]. Cuminaldehyde, the principal bioactive constituent of *Cuminum cyminum*, has been identified as a natural and highly potent inhibitor of α-synuclein fibrillation, capable of stalling β-sheet formation even under seeded aggregation conditions while markedly reducing aggregation-associated cytotoxicity without intrinsic neuronal toxicity [166]. Nordihydroguaiaretic acid (NDGA), a polyphenolic lignan with strong antioxidant, lipoxygenase inhibitory, and NRF2-activating properties, represents another BCS class IV compound with clear relevance to PD through its ability to mitigate oxidative damage and inflammatory signaling [167]. Celastrol, a bioactive triterpene, has demonstrated robust neuroprotective efficacy in PD models by restoring mitochondrial quality control, activating autophagy and mitophagy via PINK1 and DJ-1 upregulation, suppressing NF-κB–mediated neuroinflammation, modulating α-synuclein pathology, and significantly improving motor deficits in toxin-induced PD models [168,169].

A broader class of polyphenolic bioactives further underscores the depth of the formulation-limited therapeutic reservoir. Rosmarinic acid, carnosic acid, and delphinidin exhibit pleiotropic neuroprotective effects, including suppression of α-synuclein aggregation, attenuation of oxidative stress, inhibition of neuroinflammation, mitochondrial stabilization, and activation of NRF2-dependent cytoprotective pathways [170,171]. Kaempferol has gained particular attention due to its ability to directly inhibit α-synuclein fibril formation and promote autophagy–lysosomal clearance of α-synuclein via TFEB activation while also mitigating oxidative stress and inflammatory signaling through modulation of NRF2, NADPH oxidase, and TLR4-dependent pathways [172,173]. Quercetin has consistently demonstrated efficacy across multiple PD models, improving motor and non-motor behaviors, reducing oxidative burden, and suppressing pro-inflammatory cytokines such as IL-6, yet its translation remains hindered by poor solubility and permeability [174,175]. Isorhynchophylline, an indole alkaloid from *Uncaria* species, preserves nigrostriatal integrity, reduces dopaminergic neuron loss, and suppresses oxidative, endoplasmic reticulum, and mitochondria-dependent apoptotic pathways in both cellular and animal PD models [176,177].

In parallel, several repurposed synthetic drugs further strengthen the rationale for a formulation-driven strategy. Geldanamycin prevents α-synuclein aggregation and toxicity through induction of Hsp70 via Hsp90 inhibition, resulting in the preservation of dopaminergic neurons and restoration of striatal dopamine levels in PD models [178,179]. Rapamycin demonstrates disease-modifying effects by enhancing mitochondrial respiration, reducing oxidative stress, and directly inhibiting α-synuclein aggregation through FKBP12-dependent mechanisms independent of canonical autophagy [180,181]. Clioquinol targets pathological metal–α-synuclein interactions, markedly reducing iron-induced aggregation and protecting dopaminergic neurons in α-synuclein transgenic models [182,183]. Safinamide, an approved MAO-B inhibitor, provides dual dopaminergic and non-dopaminergic benefits through inhibition of MAO-B and modulation of abnormal glutamate release [184,185]. Ambroxol acts as a pharmacological chaperone for β-glucocerebrosidase, enhancing lysosomal function and reducing α-synuclein burden, particularly in GBA-associated PD. Despite their strong mechanistic alignment with PD pathogenesis, the majority of these agents belong to BCS class II or IV and have not yet been systematically explored using SNEDDSs, representing a substantial and underutilized opportunity for pathogenesis-driven drug repurposing enabled by advanced lipid-based delivery systems [186,187].

Alongside synthetic drugs and phytoconstituents, probiotics have emerged as promising adjuncts in PD therapy, showing beneficial effects across diverse experimental models. Multiple strains, including *Lactobacillus reuteri*, *Bifidobacterium breve*, *Bifidobacterium longum*, *Lacticaseibacillus rhamnosus*, *Akkermansia muciniphila*, and *Bacillus coagulans*, have demonstrated neuroprotective actions by alleviating motor and cognitive deficits, reducing oxidative stress and neuroinflammation, modulating dopamine metabolism, and restoring gut barrier integrity and microbial metabolites. These findings highlight that probiotics, through microbiota–gut–brain axis regulation, can complement synthetic and phytochemical approaches, offering a multifaceted strategy to mitigate PD progression.

Qi et al. investigated the effects of *Lactiplantibacillus plantarum* SG5 in an MPTP-induced mouse model of PD. Supplementation with SG5 alleviated motor deficits and dopaminergic neuronal injury, counteracted gut dysbiosis, and improved mitochondrial quality control in the substantia nigra by increasing PGC-1α expression and restoring mitochondrial biogenesis, dynamics, and autophagy. The protective effects were attenuated by GLP-1 receptor antagonism or PGC-1α inhibition, supporting the involvement of a gut microbiota–GLP-1/PGC-1α pathway [188]. Nevertheless, these findings remain preclinical and strain-specific and require confirmation in controlled human studies.

Aktas et al. investigated the therapeutic potential of *Lacticaseibacillus rhamnosus* E9, a high exopolysaccharide producer, which demonstrated neuroprotective effects in an MPTP-induced PD mouse model. MPTP is a neurotoxin that selectively damages dopaminergic neurons in the substantia nigra by impairing mitochondrial complex I, leading to oxidative stress and motor dysfunctions that closely mimic PD. Supplementation with E9 improved motor performance, reduced striatal ROS accumulation, modulated dopamine-related markers, and restored intestinal barrier integrity. These findings highlight E9’s therapeutic potential in mitigating MPTP-induced neurodegeneration and managing PD through gut–brain axis modulation [189].

Dong et al. demonstrated the potent activity of *Lactobacillus reuteri* to alleviate MPTP-induced PD in mice through its metabolite GABA. Elevated GABA reduced motor dysfunction and inhibited neuronal ferroptosis via the AKT-GSK3β-GPX4 pathway, highlighting *L. reuteri*-derived GABA as a promising therapeutic strategy for PD [190].

Li and colleagues explored the therapeutic potential of *Bifidobacterium longum* and demonstrated significant neuroprotective potential in Parkinson’s disease by modulating the microbiota–gut–brain axis. In MPTP-induced PD models, CCFM1029 alleviated motor dysfunction, dopaminergic neuronal loss, and neuroinflammation while restoring gut microbial balance and beneficial metabolites such as indole-3-acrylic acid and short-chain fatty acids. These findings highlight CCFM1029 as a promising probiotic candidate for preventing and mitigating PD progression through targeted microbiome regulation [191].

Valvaikar et al. studied the probiotic effect of *Bifidobacterium breve* Bif11 supplementation in a PD model and showed neuroprotective effects in MPTP-induced Parkinson’s models by improving motor and cognitive function, enhancing tyrosine hydroxylase levels, reducing neuroinflammation and oxidative stress, and restoring gut metabolites and barrier integrity. These findings suggest Bif11 as a promising probiotic candidate for mitigating PD symptoms via the microbiota–gut–brain axis [192].

Neto et al. investigated Akkermansia muciniphila for the management of PD, which is often enriched in PD fecal samples and has been shown to influence α-synuclein pathology via its conditioned medium. In vitro, the protein-enriched fraction of mucin-free CM triggered RyR-mediated Ca^2+^ release, mitochondrial Ca^2+^ uptake, ROS generation, and subsequent α-synuclein aggregation in enteroendocrine cells. Oral administration of mucin-free *A. muciniphila* induced α-synuclein aggregation in CCK-positive EECs without motor deficits, while buffering mitochondrial Ca^2+^ reversed these effects. These findings provide mechanistic evidence that bacterial proteins can initiate α-synuclein misfolding through gut–brain axis interactions [193].

Rout et al. conducted a study to attenuate rotenone-induced neurodegeneration by *Bacillus coagulans* (PBT) supplementation, demonstrating neuroprotective potential in a rotenone-induced zebrafish model of PD. Over 28 days, PBT alone and in combination with L-dopa attenuated behavioral deficits, restored dopamine levels, reduced α-synuclein aggregation, and normalized oxidative and enzymatic biomarkers (MAO-B, AChE, nitrite). Histological analysis further confirmed the prevention of neuronal damage. These findings suggest *Bacillus coagulans* as a promising probiotic candidate to mitigate PD-related neuroinflammation and neurodegeneration [194].

Ipek et al. studied the neuroprotective effect of *Saccharomyces boulardii* in a rotenone-induced rat model. When they supplemented *Saccharomyces boulardii*, bacterial probiotics, or their combination, they improved motor performance, preserved dopaminergic neurons, and reduced hippocampal degeneration compared to rotenone-only controls. Importantly, probiotics modulated gut–immune interactions, as reflected by altered CD163 expression in muscularis macrophages. These findings suggest that probiotic interventions may mitigate PD progression by preserving neuronal integrity and regulating microbiome–immune crosstalk [195].

**Figure 6:**
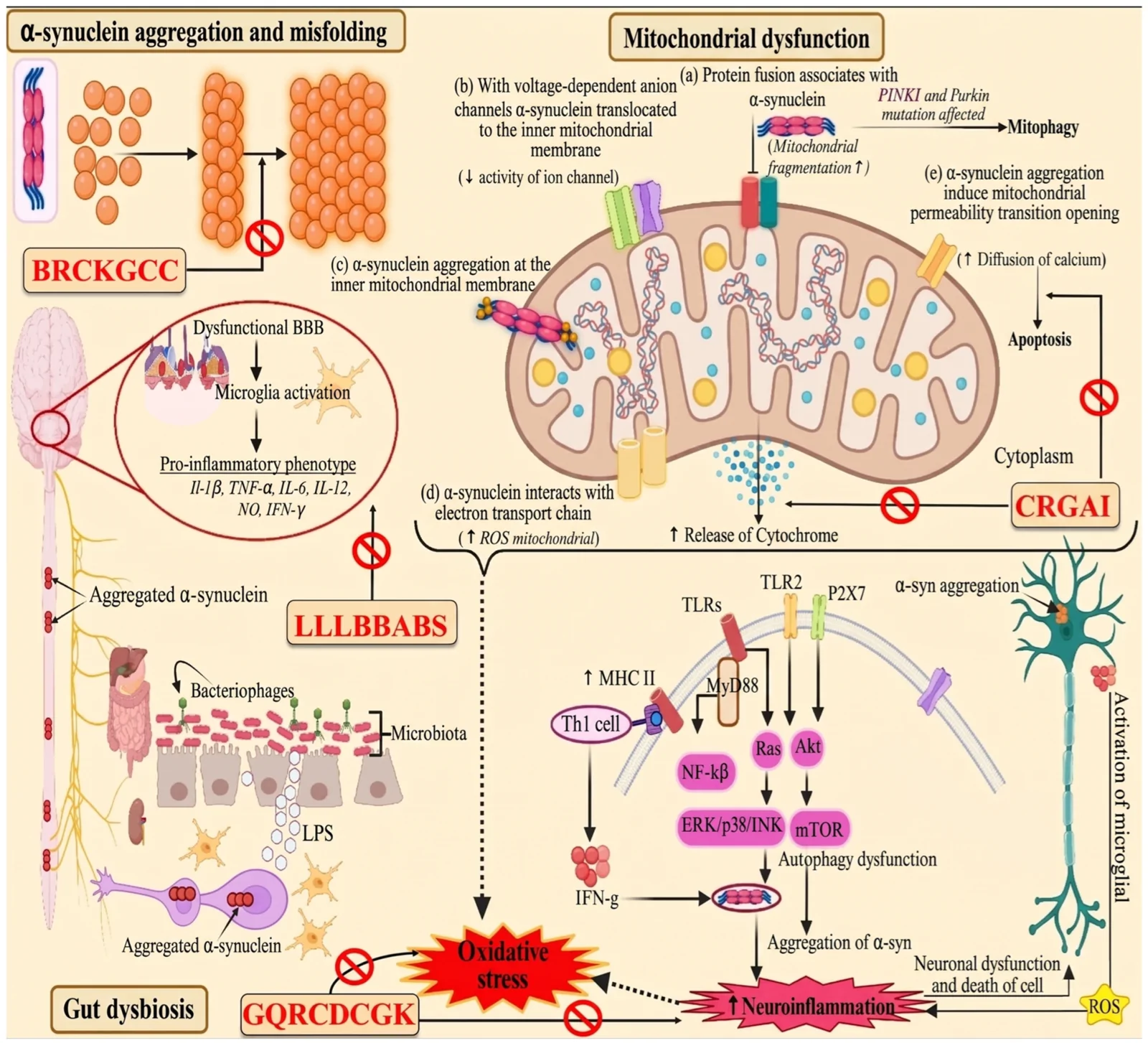
Pathogenesis-driven drug repurposing for PD. Herein, BRCKGCC targets alpha-synuclein aggregation, where B: baicalein, R: rifampicin, C: cuminaldehyde, K: Kaempferol, G: Geldanamycin, C: Clioquinol, and C: Celastrol. CRGAI for mitochondrial dysfunction inhibition drugs are C: Celastrol, R: Rapamycin, G: ginsenoside, A: ambroxol, and I: Isorhynchophylline. GQRCDCGK for oxidative stress and neuroinflammation inhibition drugs are G: gallic acid, Q: Quercetin, R: rosmarinic acid, C: carnosic acid, D: Delphindin, C: Celastrol, G: ginsenoside, and K: Kaempferol. LLLBBABS for Gut dysbiosis inhibition drugs are L: *Lactiplantibacillus plantarum*, L: *Lactobacillus rhamnous*, L: *Lactobacillus reuteri*, B: *Bifidobacterium longum*, B: *Bifidobacterium breve*, A: *Akkermansiua muciniphila*, B: *Bacillus coagulans*, and S: *Saccharomyces boulardii*.

Pathogenesis-driven drug repurposing has revealed several therapeutic agents with promising efficacy in PD, ranging from phytochemicals with antioxidant and antiaggregative properties to synthetic drugs and probiotics that modulate mitochondrial function, lysosomal activity, and the gut–brain axis. Collectively, these candidates highlight the potential to address the multifactorial nature of PD. However, despite encouraging mechanistic evidence, their translation remains constrained by poor solubility, low bioavailability, extensive first-pass metabolism, and limited CNS penetration, as illustrated in Figure 6. This is where SNEDDSs offer a rational solution: by enhancing oral solubilization, improving intestinal absorption, and facilitating lymphatic uptake, SNEDDSs can overcome many of the formulation barriers that currently limit repurposed drugs and probiotics. Looking ahead, future research should focus on systematic pharmacokinetic validation, excipient safety profiling under chronic neurodegenerative use, and early-phase clinical trials to confirm whether SNEDDS-enabled repurposing can deliver meaningful patient outcomes. If these translational hurdles are addressed, SNEDDS-based reformulation of repurposed active moieties may evolve into a next-generation therapeutic strategy for PD, combining mechanistic relevance with improved clinical feasibility.

## 9. Bottlenecks

SNEDDSs are a very promising therapeutic option to overcome various limitations of anti-Parkinson drugs. However, there are various obstacles that prevent the clinical translation of SNEDDSs. The biggest concern is dose variability since absorption is often determined by gastrointestinal physiology and food intake. This issue can be resolved to a large extent by the development of food-independent SNEDDSs that are capable of self-emulsifying efficiently under fasted conditions and by the use of absorption enhancers that can stabilize drug uptake in different GI environments. At the same time, the efficacy of SNEDDSs is largely diminished due to limited drug penetration across the BBB. Nevertheless, this problem can be solved by incorporating excipients into formulations that inhibit efflux pumps and functionalizing drugs with permeation enhancers that allow drug entry into the CNS. Moreover, the safety and toxicity of excipients are significant issues that need to be properly tackled by ensuring the in vivo biocompatibility of drugs through the correct choice of GRAS-listed excipients and natural oils. In terms of production, the main hurdle of scaling up concerns ensuring the droplet size and stability of the product; however, it is possible to get over these bottlenecks through, among other things, the use of advanced technologies like high-pressure homogenization, microfluidization, and spray-drying and the implementation of Quality by Design (QbD) approaches to ensure repeatability. At the same time, there should be strict long-term stability testing to make sure that the product is stable enough during storage and distribution.

The impact of food and patient variability, especially in elderly PD patients who have altered GI physiology, on the formulation of SNEDDSs can be mitigated by proper excipient selection and food-independent products that have low dependence on dietary lipids. In summary, these approaches represent a sound strategy that may be used to solve the main problems of SNEDDS development and to foster their use in the therapeutic management of PD.

## 10. Expert Opinion

Further studies on SNEDDSs for PD should primarily revolve around the various functionalization methods that allow for accurate targeting of the brain and dopaminergic neurons. A great potential for success lies in the reworking of SNEDDSs with ligands like transferrin, lactoferrin, or apolipoprotein E mimetics, which are capable of utilizing receptor-mediated transport over the BBB. Likewise, linking with small peptides such as TAT or RVG29 may facilitate neuronal uptake and hence drug delivery in the affected regions of the brain. Moreover, the capability of SNEDDSs to traverse the BBB can be exploited to deliver not only levodopa but also neuroprotective compounds like curcumin, resveratrol, or mitochondrial-targeting molecules, thus addressing two major factors that cause dopaminergic neuron loss: oxidative stress and mitochondrial dysfunction. Stimulus-reactive designs are also a consideration, where SNEDDSs could discharge their payload upon recognizing pathological cues, like acidic lysosomal environments or elevated ROS, thus ensuring that drug release takes place only in diseased tissues. This novel area also extends to the delivery of genes and proteins, where SNEDDSs can be modified to transport siRNA or miRNA to inhibit the genes responsible for α-synuclein aggregation, or they may encompass neurotrophic factors such as GDNF and BDNF that support neuronal survival. Surface modification, including PEGylation to extend circulation time or dual–ligand systems for higher specificity, could further enhance targeting precision, while exosome-mimicking wrappers may increase biocompatibility and the natural affinity for the brain. Although this is a transient moment of opportunity, challenges in guaranteeing the stability, scalability, and safety of these nanocarriers, as well as in navigating regulators’ ways, should not be underestimated. However, by equipping SNEDDSs with BBB-targeting ligands, neuroprotective co-therapies, stimuli-responsive release mechanisms, and genetic interventions, PD can become a playground for a versatile drug delivery platform that brings about both symptomatic relief and disease-modifying therapy. Fully embracing this perspective paves the way for SNEDDSs that increase not only the bioavailability of a drug but also the precision in targeting the complex pathophysiology of neurodegenerative diseases.

## 11. Future Perspectives

The advancement of SNEDDSs as PD formulations needs to move from empirical formula optimization to formulation approaches based on pathophysiological understanding and quantitatively validated and patient-focused delivery systems. The most urgent step in this regard would be to develop formula–pharmacokinetics–pharmacodynamics relationships by conducting appropriate studies on gastrointestinal performance, plasma pharmacokinetics, drug concentrations in the brain, pharmacodynamic effects, and relevant pathology markers in single studies. These studies will be especially essential since increased dissolution and/or bioavailability of a drug do not necessarily mean increased CNS exposure. Chronic tolerability of lipid and surfactant excipients, drug–excipient and drug–drug interactions, stability, scaling, consistency between batches, and other critical quality parameters must be assessed in the long run.

Another opportunity is the incorporation of multi-omics and precision medicine into SNEDDS development. PD shows considerable biological heterogeneity, and transcriptomic, proteomic, metabolomic, lipidomic, metagenomic, spatial, and single-cell data may be used to uncover patient-specific pathological profiles and vulnerabilities. In the future, these data may be combined with pharmacokinetic and clinical data to help select therapeutic cargo, lipid composition, surfactant system, drug loading, and drug release profiles based on the main pathological phenotype. With such an approach, SNEDDSs would be transformed from a general bioavailability enhancement tool to pathology-based oral delivery. However, this is a potential approach since clinically validated multi-omics profiles that would direct the choice of SNEDDSs in PD are not available yet.

Artificial intelligence and machine learning may further augment this strategy by building on physicochemical properties of drugs, lipids, surfactants, formulation ratios, droplet size, degree of drug loading, solubility, permeability, and stability data to predict the performance of formulations. In more advanced models, formulation parameters could be combined with PK, PD, microbiome data, and patient-specific characteristics to predict pharmacological exposure and effects. AI and explainable AI models that combine mechanisms with machine learning would be especially useful for defining the key factors in formulation behavior and for delivering explainable evidence in the formulation development and evaluation process. However, the current lack of PD-specific SNEDDS studies is a serious limitation that hinders the development of such models; thus, large datasets, external validation, and experimental confirmation will be necessary prior to reliable application of AI to formulation selection.

The gut–brain axis is another avenue for exploration. Gut permeability issues, dysbiosis, microbial metabolites, and peripheral inflammation are potential contributors to the pathogenesis of PD; meanwhile, probiotic therapy has been proven effective in selective animal and clinical trials. Therefore, further studies can explore the use of multifunctional SNEDDSs that would comprise poorly soluble neuroprotective or symptomatic agents combined with microbiome-oriented components like probiotics, postbiotics, or specific microbial metabolites. These drug delivery systems should be tested not only in terms of pharmacokinetics but also assessed by changes in gut microbial balance, intestinal permeability, short-chain fatty acids and other metabolites, systemic inflammation, α-synuclein-related pathology, neuroinflammation, and dopaminergic neuron viability. However, at this moment, probiotic-based SNEDDSs should be viewed as a hypothesis-generating technology because no direct evidence of their effectiveness in PD treatment exists yet.

An even more remote possibility may involve the emergence of theranostic and adaptive SNEDDSs. The combination of therapeutic delivery and diagnostic/biomarker sensing technologies could in theory provide the ability to monitor treatment response, biodistribution, or disease-linked biomolecular activity at the same time as the medication delivery. For instance, in PD, such an approach may, in the future, facilitate concomitant monitoring of the medication and pathology linked to neuroinflammation, oxidative stress, synuclein alterations, or dopaminergic deficit. But as traditional SNEDDSs are mainly oral solubilization and absorption systems, this is an area where many technological improvements are needed. Ultimately, clinical translation should remain the central objective rather than simply increasing formulation complexity. A staged development pathway from standardized formulation characterization and chronic safety assessment, through CNS pharmacokinetic and pharmacodynamic studies, to well-designed clinical trials comparing SNEDDSs with conventional formulations is required to determine whether the preclinical advantages observed to date translate into meaningful therapeutic benefits. Given the currently limited PD-specific clinical evidence, SNEDDSs should therefore be regarded as an emerging oral delivery platform with considerable translational potential, rather than an established clinical intervention. Future progress will depend on demonstrating that formulation-driven improvements in drug exposure translate into reproducible effects on disease-relevant biology and clinically meaningful motor and non-motor outcomes.

## 12. Conclusions

PD entails interlinked pathological processes such as α-synuclein pathology, oxidative stress, mitochondrial dysfunction, neuroinflammation, and disturbances in the gut–brain axis, thereby pointing to the necessity of developing novel strategies in addition to symptom-based therapy for this condition. SNEDDSs can serve as a reasonable platform for oral drug delivery systems capable of improving the solubility, dissolution, intestinal absorption, and bioavailability of drugs that exhibit poor solubility properties. Current preclinical research demonstrates advances in terms of formulation behavior and, in some cases, motor and biochemical effects, but it is still preclinical in nature. Combining gut dysbiosis and probiotics opens up a new angle for dealing with the gut–brain axis, whereas drug repurposing, driven by the pathophysiology of the disease, represents an opportunity to find already existing therapies and active components that can deal with certain pathological factors of PD. SNEDDSs may increase the efficacy of the delivery of these repurposed compounds by overcoming their biopharmaceutical drawbacks. But still, the combination of probiotics with SNEDDSs, as well as many other potential applications suggested by the authors, has not been sufficiently studied for PD. Further developments, including the use of multi-omics, artificial intelligence, personalized therapy, and theranostics, may help to achieve this aim, but this remains prospective.

## Figures and Tables

**Table 2:** Current FDA-approved therapies for PD: safety profiles and approval history.

Brand Name	Therapeutic Ingredients	Major Side Effect(s)	Year Approved	Reference
Sinemet	Carbidopa, Levodopa	Nausea, dyskinesia, confusion, dizziness,	1988	[84]
Rytary	Carbidopa, Levodopa (extended-release)	Nausea, dyskinesia, hallucinations	2015	[85]
Stalevo	Carbidopa, Levodopa, Entacapone	Urine discoloration, diarrhea, dyskinesia, confusion,	2003	[86]
Comtan	Entacapone	Abdominal pain, diarrhea, nausea, and urine discoloration	1999	[87]
Ongentys	Opicapone	Constipation, dyskinesia, low blood pressure, insomnia	2020	[88]
Tasmar	Tolcapone	Liver toxicity, dizziness, diarrhea, and sleep disturbance	1998	[89]
Azilect	Rasagiline	Dizziness, headache, joint pain, depression, indigestion	2006	[90]
Eldepryl	Selegiline	Nausea, insomnia, dizziness, headache	1989	[91]
Xadago	Safinamide	Uncontrolled movements, insomnia, nausea, falls	2017	[92]
Apokyn	Apomorphine	Low blood pressure, nausea, vomiting, sleepiness, site reactions	2004	[93]
Neupro	Rotigotine (skin patch)	Drowsiness, nausea, hallucinations	2007	[94]
Mirapex	Pramipexole	Sleepiness, hallucinations, nausea	1997	[95]
Trihexyphenidyl	Trihexyphenidyl	Dry mouth, constipation, blurred vision, confusion	2003	[96]
Cogentin	Benztropine	Dry mouth, confusion, blurred vision, urinary retention	1954	[96]
Gocovri	Amantadine (ER)	Confusion, hallucinations, edema, livedo reticularis	2017	[97]
Exelon	Rivastigmine	Nausea, vomiting, loss of appetite, weight loss	2000	[98]
Nuplazid	Pimavanserin	Swelling, hallucinations, confusion	2016	[99]
Nourianz	Istradefylline	Dyskinesia, hallucinations, constipation, insomnia	2019	[100]

## Data Availability

No new data were created or analyzed in this study. Data sharing is not applicable to this article.

## Abbreviations

The following abbreviations are used in this manuscript:

AD	Alzheimer’s Disease
AQP4	Aquaporin-4
BBB	Blood–Brain Barrier
BCS	Biopharmaceutics Classification System
CNS	Central Nervous System
COMT	Catechol-O-Methyltransferase
CSF	Cerebrospinal Fluid
FDA	Food and Drug Administration
GI	Gastrointestinal
HIF-1α	Hypoxia-Inducible Factor-1 Alpha
IL	Interleukin
ISF	Interstitial Fluid
LDL	Low-Density Lipoprotein
LLPS	Liquid–Liquid Phase Separation
LPS	Lipopolysaccharide
LCTs	Long-Chain Triglycerides
MAO-B	Monoamine Oxidase-B
MCTs	Medium-Chain Triglycerides
MDS-UPDRS	Movement Disorder Society–Unified Parkinson’s Disease Rating Scale
NF-κB	Nuclear Factor Kappa-B
PD	Parkinson’s Disease
PEG	Poly(Ethylene Glycol)
PINK1	PTEN-Induced Kinase 1
PLGA	Poly(Lactic-co-Glycolic Acid)
ROS	Reactive Oxygen Species
SCFAs	Short-Chain Fatty Acids
SNc	Substantia Nigra Pars Compacta
SNEDDS	Self-Nanoemulsifying Drug Delivery Systems
TLR	Toll-Like Receptor
TNF-α	Tumor Necrosis Factor-Alpha
